# Burden of Rare Variants in ALS and Axonal Hereditary Neuropathy Genes Influence Survival in ALS: Insights from a Next Generation Sequencing Study of an Italian ALS Cohort

**DOI:** 10.3390/ijms21093346

**Published:** 2020-05-08

**Authors:** Stefania Scarlino, Teuta Domi, Laura Pozzi, Alessandro Romano, Giovanni Battista Pipitone, Yuri Matteo Falzone, Lorena Mosca, Silvana Penco, Christian Lunetta, Valeria Sansone, Lucio Tremolizzo, Raffaella Fazio, Federica Agosta, Massimo Filippi, Paola Carrera, Nilo Riva, Angelo Quattrini

**Affiliations:** 1 Experimental Neuropathology Unit, Institute of Experimental Neurology (INSPE), Division of Neuroscience, San Raffaele Scientific Institute, 20132 Milan, Italy; scarlino.stefania@hsr.it (S.S.); domi.teuta@hsr.it (T.D.); pozzi.laura@hsr.it (L.P.); alessandro.romano@hsr.it (A.R.); yuri.falzone@hsr.it (Y.M.F.); quattrini.angelo@hsr.it (A.Q.); 2 Laboratory of Clinical Molecular Biology, Unit of Genomics for Human Disease Diagnosis, Division of Genetics and Cell Biology, San Raffaele Scientific Institute, 20132 Milan, Italy; pipitone.giovanni@hsr.it (G.B.P.); carrera.paola@hsr.it (P.C.); 3 Neurology Unit, San Raffaele Scientific Institute, 20132 Milan, Italy; fazio.raffaella@hsr.it (R.F.); filippi.massimo@hsr.it (M.F.); 4 Medical Genetic Unit, Department of Laboratory Medicine, Niguarda Hospital, 20132 Milan, Italy; lorena.mosca@ospedaleniguarda.it (L.M.); penco.silvana@ospedaleniguarda.it (S.P.); 5 NEuroMuscular Omnicentre (NEMO), Fondazione Serena Onlus, Milan 20132, Italy; christian.lunetta@centrocliniconemo.it (C.L.); valeria.sansone@centrocliniconemo.it (V.S.); 6 Department Biomedical Sciences of Health, University of Milan, 20132 Milan, Italy; 7 Neurology Unit, “San Gerardo” Hospital and University of Milano-Bicocca, 20900 Monza, Italy; lucio.tremolizzo@unimib.it; 8 Neuroimaging Research Unit, Institute of Experimental Neurology (INSPE), Division of Neuroscience, San Raffaele Scientific Institute, 20132 Milan, Italy; federica.agosta@hsr.it; 9 Neurophysiology Unit, IRCCS San Raffaele Scientific Institute, 20132 Milan, Italy; 10 Vita-Salute San Raffaele University,20132 Milan, Italy

**Keywords:** motor neuron disease, lower motor neuron syndrome, nerve, neuropathy, CMT, distal SMA, hereditary neuropathy, genetics, survival, next generation sequencing

## Abstract

Although the genetic architecture of amyotrophic lateral sclerosis (ALS) is incompletely understood, recent findings suggest a complex model of inheritance in ALS, which is consistent with a multistep pathogenetic process. Therefore, the aim of our work is to further explore the architecture of ALS using targeted next generation sequencing (NGS) analysis, enriched in motor neuron diseases (MND)-associated genes which are also implicated in axonal hereditary motor neuropathy (HMN), in order to investigate if disease expression, including the progression rate, could be influenced by the combination of multiple rare gene variants. We analyzed 29 genes in an Italian cohort of 83 patients with both familial and sporadic ALS. Overall, we detected 43 rare variants in 17 different genes and found that 43.4% of the ALS patients harbored a variant in at least one of the investigated genes. Of note, 27.9% of the variants were identified in other MND- and HMN-associated genes. Moreover, multiple gene variants were identified in 17% of the patients. The burden of rare variants is associated with reduced survival and with the time to reach King stage 4, i.e., the time to reach the need for percutaneous endoscopic gastrostomy (PEG) positioning or non-invasive mechanical ventilation (NIMV) initiation, independently of known negative prognostic factors. Our data contribute to a better understanding of the molecular basis of ALS supporting the hypothesis that rare variant burden could play a role in the multistep model of disease and could exert a negative prognostic effect. Moreover, we further extend the genetic landscape of ALS to other MND-associated genes traditionally implicated in degenerative diseases of peripheral axons, such as HMN and CMT2.

## 1. Introduction

Amyotrophic lateral sclerosis (ALS), also known as Lou Gehrig’s disease, is the most frequent and severe disease within the widely heterogeneous spectrum of adult-onset motor neuron diseases (MND). It is defined by the degeneration of both upper motor neurons (UMN) and lower motor neurons (LMN) in the cerebral cortex, brain stem, and spinal cord [1,2]. This leads to progressive muscle weakness, wasting, and rapidly progressive paralysis which typically causes death due to respiratory failure within three to five years after the symptom onset [1]. About 10% of ALS cases are familial (fALS), whereas the remaining 90% apparently occur sporadically (sALS) [3,4]. To date, more than 30 genes have been reproducibly implicated in ALS, while more than 120 have been proposed as potentially related to ALS, although most are still of uncertain and often not replicated significance [1,2]. In about 60–80% of fALS patients, a gene mutation can be identified, with the four major ALS genes by frequency being *SOD1*, *C9orf72*, *TARDBP,* and *FUS* [1,2,4]. While less well understood, the genetic hereditability of sALS has been estimated to be more than 60% [5] and the contribution of rare variants with intermediate to large effects is increasingly recognized in the context of an oligogenic model of the disease [2,6,7]. According to this hypothesis, each co-occurring variant alone could be tolerated but when combined with a second variant would exceed the threshold required for neurodegeneration [8]. However, genetic heterogeneity, pleiotropy, and nonpenetrance are incompletely understood issues in ALS [1,2,4,6]. Furthermore, some aspects of the ALS phenotype, are shared with other neurodegenerative, neuropsychiatric, and neuromuscular diseases, such as axonal hereditary Charcot-Marie-Tooth neuropathy (CMT2), distal hereditary motor neuropathy (dHMN), or hereditary spastic paraplegia (HSP), which could implicate similar genes in their pathology [1,2,9,10,11].

The aim of our work was to further explore the architecture of ALS by using a next generation sequencing (NGS) panel, specifically enriched in ALS and other non-ALS, MND-associated genes, involved in HMN, CMT2, and HSP, in order to investigate if disease expression, including progression rate, could be influenced by the combination of multiple rare gene variants.

## 2. Results

### 2.1. Variant Identification and Classification

The 83 study participants (Table S1) were analyzed with NGS, Sanger direct sequencing, and *C9orf72* analysis. Overall, we detected and confirmed, with the Sanger technique, 43 rare or novel variants in coding or splice site regions in 17 different genes, which satisfied the predefined filter criteria.

Of the variants identified, 31 (72%) were detected in ALS genes and 12 (28%) in CMT2 and dHMN genes. Thirty-six patients (43.4% overall, 47% in fALS and 42% in sALS) carried at least one of these variants. Overall, with regard to NGS analysis, the variants distribution was as follows: 90.4% missense, 2.4% nonsense, 4.8% frameshift, and 2.4% splice site.

**Figure 1:**
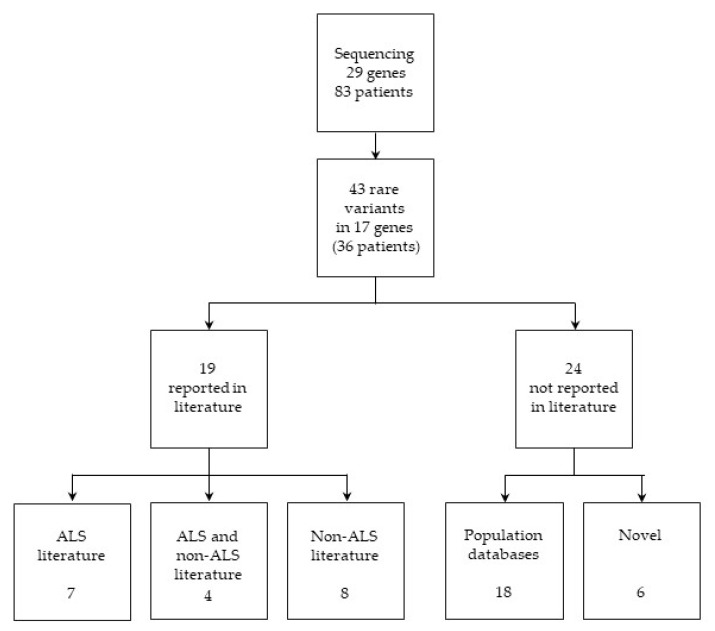
Variant identification and classification.

Among the detected variants, 44% of the detected variants (*n* = 19) were previously described in the literature: seven of them were reported in the ALS literature, four in the ALS and non-ALS literature, and eight in the non-ALS literature. The remaining 56% of the variants (*n* = 24) were not reported in the literature; 18 were annotated only in population databases, of which one only in the Single Nucleotide Polymorphism Database (dbSNP), while six were novel and could be considered as candidate ALS-associated variants (Figure 1 and Table 1). Rare variants, as well as clinical and demographic data of individual patients are reported in Table S2. Rare variants identified in a group of 332 non-neurological unrelated Italian patients, for whom NGS exome sequencing data were available from our in-house database, were selected as the control group (Tables S3–S5).

#### 2.1.1. Variants Previously Reported in ALS Literature

Seven patients were harboring the pathogenic *C9orf72* repeat expansion. Three previously reported missense variants were classified by the American College of Medical Genetics and Genomics (ACMG) criteria as pathogenic as follows: the *SOD1* p.Leu68Pro and p.Gly73Ser, and the *TBK1* p.Ile397Thr. Among them, the two *SOD1* variants were described in both fALS and sALS cases [12,13] and in an Italian sALS case, respectively [14,15]. The *TBK1* p.Ile397Thr was previously described by our group [16]. Three variants, reported in previous NGS studies, were classified as variants of uncertain significance (VUS) as follows: the *ALS2* p.Pro372Arg, the *OPTN* p.Gln314Leu, and the *SETX* p.Lys218Asn [15,17,18]. Notably, we identified the *BSCL2* p.Leu427Pro missense variant, previously reported in ALS literature [18], in three unrelated patients but also in nine controls, hence, this variant was excluded from this study (Tables S3 and S4).

#### 2.1.2. Variants Previously Reported in both ALS and Non-ALS Literature

Four variants, which were identified in seven different ALS patients, have been previously reported in both the ALS and non-ALS literature. We identified the *SETX* p.Arg20His variant, classified as VUS, in three unrelated patients, presenting with a pyramidal, predominant upper motor neuron (PUMN), and classic phenotype, respectively. This variant has previously been identified in compound heterozygosity in patients with ataxia with oculomotor apraxia type 2 (AOA2) [19] and in patients with CMT2 [20]; however, it has also been previously reported in ALS individuals [8,15,21]. The three variants identified by our screening in the *SPG11* gene have previously been reported in both ALS [15,18] and hereditary spastic paraplegia (HSP) cohorts [22]. We also identified the following two variants in ALS patients and control cases, classified by ACMG as likely pathogenic: the *FIG4* p.Ile41Thr and the *ERBB4* p.His374Gln (Table S4). The *FIG4* p.Ile41Thr missense, originally described in compound heterozygous in demyelinating autosomal recessive CMT neuropathy type 4 juvenile (CMT4J) patients [23,24,25,26], was also subsequently reported in ALS patients, with autosomal dominant transmission [8,18,27,28]. The *ERBB4* p.His374Gln missense, described in ALS [29] and cancer patients [30], was identified in one sALS and one unrelated fALS patient. These variants were excluded from our results.

#### 2.1.3. Variants Previously Reported in Non-ALS Literature

We identified eight variants that have been previously reported in the non-ALS literature. Among them, seven variants have previously been associated with a neurological phenotype, while three variants have been associated with patients with non-neurological diseases. The *SPG11* p.Cys1996Leufs*4 frameshift was classified by ACMG as pathogenic. This variant has been previously reported in compound heterozygosis in one Israeli [31] and one Italian patient [32], both presenting with autosomal recessive (AR) HSP. Similarly, we identified this variant in a classic sALS patient with *SPG11* compound heterozygosity. The following six variants were classified as VUS: the *MFN2* p.Asn525Ser. the *BSCL2* p.Ala282Thr and p.Arg345Trp. the *SETX* p.Arg1538Trp. and the *SQSTM1* p.Leu268Val and p.Pro118Ser. The *MFN2* p.Asn525Ser, which was identified in a bulbar sALS patient, has been previously reported in a large NGS CMT study [33]. The *SQSTM1* p.Leu268Val has been previously reported in one Italian patient with Alzheimer’s disease (AD) [34]; additionally, we identified another *SQSTM1* variant (p.Pro118Ser), which had been previously reported in one patient with ALS, a frontotemporal dementia (FTD) patient, and in two control individuals [34,35]. The *BSCL2* and *SETX* VUS variants have been previously reported in B-cell lymphoma [36] and cancer patients, respectively [37]. Finally, the *HSPB3* p.Arg116Pro variant has recently been described in myopathy patients [38]. This variant is predicted to be deleterious by in silico programs, since it is located in the highly conserved (alfa) crystalline domain [38].

The following four variants were identified in our in-house controls and therefore were excluded from our results: the *ALS2* Gly1069Glu, the *MFN2* p.Arg468His, the *SPG7* p.Ala510Val, and p.Arg486Gln variants. The *ALS2* Gly1069Glu, which was identified in a pyramidal sALS patient, has been reported in the HSP family by a previous NGS study [39]. We identified the *MFN2* p.Arg468His variant in a patient with flail arm syndrome, a restricted ALS phenotype characterized by predominant LMN involvement in upper limbs, and pes cavus (i.e., an abnormally high plantar longitudinal arch). This variant, whose pathogenicity has also been supported by functional in vitro studies [40], has been previously reported in patients with axonal CMT (CMT2) [41,42,43], although a role in demyelinating CMT (CMT1) has been more recently suggested [44,45]. The *SPG7* p.Ala510Val variant has been previously reported in patients with AR HSP [46,47], whereas the p.Arg486Gln has been previously reported in both AR HSP and progressive external ophthalmoplegia (PEO) [48,49,50]. The *SPG7* p.Ala510Val variant is located in a highly conserved region and its pathogenicity has been previously supported in a yeast model [51].

#### 2.1.4. Variants Present Only in Population Databases

Eighteen variants were not reported in the literature but were recorded in the general population as variants at a very low frequency and have not been previously associated with a clinical phenotype. *PLEKHG5* p.Gly1017Arg was reported only in dbSNP. We identified five patients harboring three different variants in the *BSCL2* gene. Among these variants, the p.Ser230Asn variant, identified in a bulbar sALS patient, was predicted as potentially pathogenic by ACMG classification, while the p.Ser353Thr and the p.Pro428Ser were classified as VUS and were both found in patients harboring more variants of interest. The *DCTN1* p.Val496Leu variant, which was located in the dynein binding domain and predicted as pathogenic, was identified in a fALS patient presenting with a classic phenotype and no cognitive impairment. Notably, autosomal dominant (AD) *DCTN1* mutations have been reported in patients presenting with a lower motor neuron disease, ALS, and related to the ALS and FDT families [52,53]. While mutations in the *HSPB1* gene have been previously associated with HMN, dHMN, and CMT2 [54,55], we detected the *HSPB1* p.Ser135Ala in a female patient presenting with a classic ALS phenotype. This variant is located in the protein alpha-crystalline domain and is classified as likely pathogenic by ACMG. The *HSPB3* p.Arg116X, predicted to create a premature stop codon, and thus classified as likely pathogenic, was identified in a patient presenting with classic ALS associated with FTD. The *HSPB3* p.Gly67Ser was instead classified as VUS. Finally, the *SPG11* p.Ser559Thr which was identified in a pyramidal sALS patient and the three *DYNC1H1* variants, a gene previously associated with AD CMT2 and spinal muscular atrophy (SMA) phenotypes [56], were all classified as VUS. The *PLEKHG5* p.Arg107Cys was identified in two unrelated sALS patients and controls, and therefore was excluded from the study.

#### 2.1.5. Novel Candidate ALS-Associated Variants

Six variants were absent from all public genomic databases, including dbSNP, and can be considered to be novel. Of the two novel *DYNC1H1* variants, the p.Lys1395Gln was predicted as likely pathogenic. Interestingly, this variant was detected in a fALS patient harboring multiple gene variants and presenting with an aggressive disease course and 11 months survival. The *DYNC1H1* p.Ser1768Ile was instead predicted as VUS. The *SPG11* frameshift p.Met1609Serfs*31 was the only one identified in a patient presenting with a flail arm phenotype associated with FTD. The *FIG4* p.Pro344Thr variant, identified in a flail arm patient, was located in the protein phosphatase domain and was predicted as likely pathogenic by ACMG classification. Finally, the *SETX* p.His1951Leu variant was identified in a patient with multiple variants. Further investigations are required to determine if the novel variants have an effect at the protein level, both in the structure or in the functional activity, as well as in in affected individuals and pedigrees.

### 2.2. Co-Occurrence of Variants in ALS Genes Panel and Survival Analysis

In our cohort of ALS patients, 14 patients (17% overall, 24% in fALS, 15% in sALS) harbored multiple rare variants in more than one gene. Eleven of these fourteen patients presented changes in two genes and three patients carried changes in three genes (Table 2). Thirteen of these fourteen patients (93%) showed at least one variant in an ALS-associated gene; three patients harbored changes in three ALS genes, four patients in two ALS genes, while six patients harbored one gene variant in an ALS gene associated with one variant in a HMN/CMT2 gene. Finally, one patient had two variants in MND-spectrum genes. Interestingly, the two patients with the previously reported mutations in the *SOD1* gene presented changes in another ALS gene, while four of the seven patients with the pathogenic *C9orf72* repeat expansion were harboring a second gene variant. The *C9orf72* repeat expansion was associated with ALS associated genes (*DCTN1* and *DYNC1H1*) and MND-spectrum genes (*HSPB1*); of note, all these genes were previously associated with a dHMN, HMN, or CMT2 phenotype.

**Figure 2:**
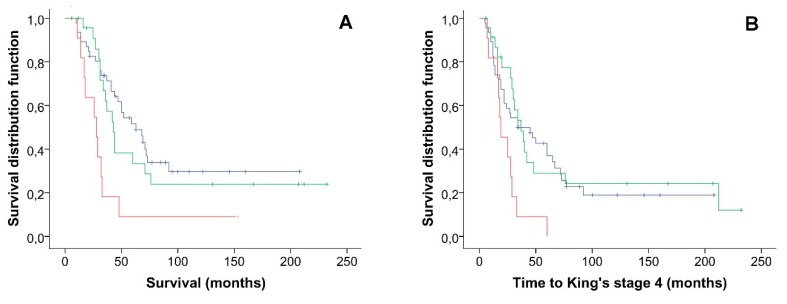
Kaplan–Meier univariate analysis for Survival (**A**) and time to reach King stage 4 (**B**). Black line, patients harboring no variants; green line, one rare variant; red line, two or more rare variants.

The Kaplan–Meier analysis showed that a higher variant burden is associated with reduced survival in our cohort of ALS patients, log-rank (Mantel–Cox) χ^2^ = 10.52, *p* = 0.005 (Figure 2A). The ALS patients harboring two or more rare variants had a significantly shorter median survival (28.00 months, 95% CI 16.1–39.9 months) as compared with the ALS patients carrying one rare variant (43.00 months, 95% CI 35.1–50.8) and with the ALS patients harboring no rare variants (63.00 months, 95% CI 41.8–84.1 months). Additionally, we showed that a higher rare variant burden is associated with reduced time to reach King stage 4, log-rank (Mantel–Cox) χ^2^ = 8.61, *p* = 0.01 (Figure 2B) as compared with the ALS patients harboring one or no rare variants. The ALS patients harboring two or more rare variants have a significantly shorter median time to reach King stage 4 (19.00 months, 95% CI 10.4–27.6 months) as compared with the ALS patients carrying one rare variant (37.00 months, 95% CI 25.2–48.8) and with the ALS patients harboring no rare variants (34.00 months, 95% CI 10.1–57.9 months). These results were confirmed at MAF < 0.001 in the European population of the ExAC database (Figure S1). The Kaplan–Meier analysis did not show an association between rare variant burden and age at onset.

The Cox multivariate analysis confirmed that the burden of rare variants is independently associated with reduced survival in MND with an increased proportional hazard ratio (HR) for patients harboring more than two rare variants of 5.61 (95% CI 2.38 to 13.22, *p* ≤ 0.001) (Table 3). Similarly, the Cox regression model confirmed that the burden of rare variants is an independent prognostic factor for the time to reach Kings stage 4, with an increased HR for patients harboring more than two rare variants of 3.09 (95% CI 1.37 to 6.97, *p* = 0.007). Both results were confirmed at MAF < 0.001. (Table S6).

## 3. Discussion

In this study, by using next generation sequencing and *C9orf72* repeat expansion analysis, we analyzed 29 genes in an Italian cohort of 83 patients with both familial and sporadic ALS. Overall, we detected 43 rare variants in 17 different genes and found that 43.4% of ALS patients harbored a variant in at least one of the investigated genes, with 17% of the patients showing co-occurrence of more than one gene variant. Moreover, our results show that rare variant burden is associated with reduced survival and the time to reach King stage 4, independently of known negative prognostic factors.

Overall, our findings confirm and highlight the importance of genetic susceptibility in both familial ALS, as well as in a major proportion of apparently sporadic ALS cases [15,17,57]. A direct comparison with previous studies is methodologically difficult, since our cohort was clinic-based with no strict inclusion criteria and we could not address whether differences in populations could be involved. However, the high frequency of variants detected in our cohort could probably, in part, be due to the large number of genes we examined and, in part, because some variants were observed only in a heterozygous form but could be associated with ALS as recessive or within the context of a multistep disease model [6]. Moreover, apparently, sALS patients can carry pathogenic variants with low penetrance influencing their classification as fALS versus sALS [8,17].

An interesting finding is that 28% of the variants were identified in CMT2/dSMA genes. These data confirm the emerging genetic pleiotropy of ALS [1,2,9,58]. More specifically, in relation to the specific design of our panel, our data suggest an overlap with other diseases sharing degeneration of motor axons and neurons as a common feature, such as the axonal hereditary neuropathies [9].

Mutations in the *MFN2* gene are largely considered to be the primary cause underlying CMT2 [59]. Pes cavus, which is traditionally considered a distinguishing CMT clinical sign, has also been anecdotally reported in ALS patients [60], and is estimated to be as high as 2% of our case series [8]. We identified two *MFN2* variants in our cohort. The p.Arg468His variant has been previously reported in CMT2 patients and its pathogenicity has also been supported by functional in vitro studies [33]. However, we excluded this variant since it was identified in our non-neurological control group.

Mutation in *DYNC1H1*, classified by the ALSoD database as an ALS gene, have been previously associated with autosomal dominant CMT type 2O but also with spinal muscular atrophy with a predominance of lower extremity involvement (SMA-LED), HSP, and hereditary mental retardation with cortical neuronal migration defects [59,60,61]. Among the five variants identified by our screening in *DYNC1H1*, the p.Lys1395Gln, which was predicted as pathogenic by in silico tools, was interestingly detected in a fALS patient with multiple gene variants and presenting with a classic ALS and aggressive disease.

Mutations in *BSCL2*, encoding Seipin, are responsible for pleiotropic clinical manifestations ranging from autosomal-recessive congenital generalized lipodystrophy to autosomal-dominant seipin-related motor neuron diseases, such as distal hereditary motor neuropathy type V (dHMN-V), Silver syndrome/SPG17, and CMT2 [62]. Among the variants we found in this gene, three were already reported as VUS, while the other two variants, p.Ser230Asn and p.Ser353Thr, were present in ExAC database at a very low frequency (MAF < 0.00001). Among them, the former was located in the highly conserved functional domain, analogously to the p.Ala282Thr identified by us, while the Ser353 was a cAMP and cGMP-dependent protein kinase phosphorylation site, which allowed us to speculate about the potential pathogenicity of the latter variant [63].

Structurally, small heat shock proteins B (HSPBs) are characterized by a C-terminal α-crystallin domain, which is highly conserved and important for the formation of stable dimers; therefore, mutations involving this sequence are regarded as disease causing [64]. To date, about twenty different mutations have been reported in the *HSPB1* gene, associated with HMN, dHMN, dSMA, and CMT2, with both dominant and recessive inheritance [54,55]. We found the novel p.Ser135Ala variant, located in the α-crystalline domain and predicted as damaging by in silico tools. *HSPB1* p.Ser135Phe/Cys/Tyr mutations have already been reported in dHMN and CMT2, [54,55] supporting the pathogenicity of this new variant. Intriguingly, our patient presented with flail leg syndrome, a restricted ALS phenotype characterized by predominant LMN involvement in the lower limbs and was also harboring the pathogenic *C9orf72* repeat expansion. *HSPB3*, a paralogue of *HSPB1,* is expressed in several tissues including adult smooth muscle and brain [65]. Several mutations in this one-exon gene have been previously associated with distal hereditary motor neuropathy 2C (dHMN2C), myopathy, and CMT2 [64]. The p.Arg116Pro mutation which we found in this gene has recently been described in a patient with myopathy and functional studies have provided evidence that *HSPB3* mutants could be associated with a gain of toxic function by inhibiting the formation of the HSPB2-HSPB3 complex and increasing the propensity to form nuclear aggregates [38]. Notably, in the same position, we identified the p.Arg116X nonsense variant, never reported before in the literature, in a patient with a classic phenotype. Therefore, this variant could be considered to be likely pathogenic.

Although *FIG4* is classified as a major ALS gene, it was originally reported as a causative gene for CMT4J, an AR motor and sensory neuropathy [23,27]. The *FIG4* p.Pro344Thr variant, identified in a flail arm patient, is located in the protein phosphatase functional domain and is predicted as deleterious by in silico tools; hence, further studies are needed to understand if this alteration can influence the protein activity. Although the same p.Ile41Thr mutation described first in CMT4J was also subsequently identified in AD fALS and sALS [28], we also found it in two controls, not supporting its pathogenicity.

In the *SETX* gene, we found nine unrelated patients who were carrying three variants classified as VUS, which have been reported in the ALS and non-ALS literature, including CMT2 and AOA2 [19,20], as well as the novel p.His1951Leu variant which was identified in a patient with multiple gene variants. Interestingly, as already noticed in prior NGS studies, we found an abundance of variants in this gene, which are generally described as rare [8,15].

Among the variants identified in ALS genes, we found an in-frame deletion in the protein glycin-rich domain of the *FUS* gene, which is absent from all public genomic databases or dbSNP. Similar insertions and deletions of G-residues in this G10-stretch region have already been reported in ALS patients [66], but also in control individuals, as confirmed by the analysis of our control group, suggesting that small G-deletions in this region could be tolerated [67,68].

In spite of the relative abundance of *SPG11* variants, this gene is associated with AR HSP with thin corpus callosum and juvenile ALS under recessive disease models [69,70]. We identified the p.Cys1996Leufs*4 frameshift in a classic sALS patient with *SPG11* compound heterozygosity; intriguingly, this variant has been previously reported as pathogenic in compound heterozygosity in two different unrelated patients, however, presentin with an HSP phenotype [31,32].

Recent findings have suggested a complex model of inheritance in ALS, which is consistent with a multistep pathogenetic process and could explain phenomena such as reduced penetrance or phenotypic heterogeneity, and clinical presentation and progression, which can be observed even in familial cases and between patients harboring the same gene mutation [6,8]. In our cohort, about 15% of patients were harboring variants in at least two of the investigated genes, confirming the findings of previous studies and supporting the hypothesis that rare variant burden can play a role in the multistep model of disease. Such a model would be inclusive of patients with mutations in major ALS genes [8,15,17,57,71,72,73], even if for such genes as *C9orf72*, *SOD1*, *TARDBP* and *FUS* a reduced number of pathogenetic steps has been recently suggested [74]. It is important to note that the percentage of co-occurrence of gene variants estimated by our study is higher as compared with most of the previous studies, including a recent large-scale study, identifying oligogenity in 1% of ALS patients [75]. The reported frequencies of patients with multiple variants in ALS are indeed varied, having been estimated in previous studies as 1.6% [15], 1.3% [76], 1.4% [72], 3.8% [8], 7.9% [73], 18.8% [17], and 19.5% [71]. These differences reflect a number of methodological issues such as the number and selection of sequenced genes (i.e., gene size effect or genetic variability of studied genes), presence of controls, and criteria adopted for filtering and, most importantly, for predicting the pathogenicity of identified variants. Indeed, there is no consensus on the criteria for defining a pathogenic combination of mutations, and hence the appropriate use of the “oligogenic inheritance” term itself is debatable since some variants can be classified as VUS [77] and the co-occurrence of a pathogenic variant with a VUS has been considered to be oligogenic inheritance by some studies [17,71,72], whereas in other studies the term was rigorously restricted to previously reported or potentially pathogenic variants [75]. Therefore, we acknowledge that further mechanistic functional analyses or segregation studies are needed in order to scrutinize the pathogenicity of each of the variants we identified. Indeed, the various in silico models for prediction of pathogenicity are not always consistent (Table 1) [77]; for this reason, we treated all rare variants equally in the survival analysis and did not take into account whether some variants could be more deleterious than others, in line with previous reports [17,71,72]. Notably, we showed that a higher variant burden is associated with reduced survival and time to reach King stage 4, i.e., the time to reach such important disease milestones as the need for percutaneous endoscopic gastrostomy (PEG) positioning or non-invasive mechanical ventilation (NIMV) initiation, independently from known negative prognostic factors [78,79]. This finding supports a model of ALS where the additive or synergistic effects of multiple defective genes influence disease phenotype and, in particular, affects the progression of ALS. Therefore, these data could potentially have important implications not only in clinical practice, but also for patient stratification in clinical trials, using their genetic profile.

Although an association between rare variant burden and the time to reach King stage 4 has never been explored before, our data are in accordance with previous studies, similarly showing a reduced survival in patients with variants in more than one gene [71] and a correlation with the rate of disease progression [80]. Our results did not show a relation between rare variant burden and age of onset, in line with previous reports [68]. However, other papers reported conflicting results, suggesting either earlier [8,73] or later [17] age of onset in patients carrying variants in more than one ALS gene.

Taken together, these data point to an emerging relevance of oligogenic models in ALS with growing implications in ALS molecular diagnosis and counseling, genotype–phenotype correlation studies, as well as the development of novel therapeutic strategies.

Our study has several limitations. First, our sample size is relatively small, and this is not a population-based study and as such it is potentially affected by referral bias. However, we were able to provide detailed phenotyping in order to test potential genotype–phenotype correlations. Secondly, unlike most of previous studies, although we included in our analysis some genes associated with HMN/dSMA/CMT2, based on their association with other hereditary motor syndromes [9], we acknowledge that the gene list is far from being exhaustive, and therefore this is not a truly unbiased survey of the exome or genome. However, our study design allowed us to explore specific hypotheses. We also acknowledge that the panel was designed before the discovery of some more recent ALS genes. The development of NGS technologies has certainly given us the advantage of accelerating the generation of a large amount of sequencing data; however, it has also emphasized the complexity of determining the pathogenicity of variants. This can be even more difficult in complex diseases such as ALS, for which a multistep pathogenetic process has been proposed [6]. Indeed, individual variants alone could be tolerated but when combined with a second variant would exceed the threshold required for neurodegeneration [8], a consideration that led us to not restrict our analysis to previously reported or pathogenic ALS variants, in line with previous studies [17,71,72], even if other studies adopted more stringent criteria [75]. Nonetheless, it could also be anticipated that many of the possible disease variants identified by NGS studies are not associated with ALS [15]. Indeed, it has been shown that individuals from different populations carry different profiles of rare and common variants, and that low-frequency variants show substantial geographic differentiation [81]. Given that variants in a certain region or domain of a gene are associated with ALS as disease causing variants are rare, it could be possible that the lack of study of specific populations could explain the number of variants that have not been previously reported. Moreover, the results obtained could be linked to the size of the gene or the intrinsic variability of the genes tested. In order to control for these biases, we analyzed a large cohort of in-house non-neurological controls, and excluded the variants found in the two groups. However, our analysis of non-neurological controls showed that the gene variability was comparable to that observed in the ALS group and some variants that were associated with ALS by previous studies could also be detected in the controls (Table S5), which is an observation in line with previous reports [75]. In support of our results, however, our survival analysis showed an independent negative effect for patients harboring more than one variant, indirectly corroborating the hypothesis of a potential detrimental effect of the burden of the rare variants that we identified. However, as stated above, we acknowledge that many of the novel and rare variants identified require further studies in larger cohorts of patients to validate a possible contribution to ALS.

In summary, our data contribute to a better understanding of the molecular basis of ALS supporting an oligogenic model of disease and a negative prognostic effect of rare variant burden. Moreover, our results suggest a further extension of the genetic landscape of ALS to other genes traditionally implicated in degenerative diseases of peripheral axons, such as HMN, dSMA, and CMT2. However, our results should be replicated in other independent and larger cohorts of ALS patients.

## 4. Materials and Methods

### 4.1. Subjects

We sequenced a cohort of 83 unrelated Italian ALS patients. The local ethics committee approved the study protocol. No strict inclusion or exclusion criteria were adopted; patients were included upon clinical judgment; individuals with earlier disease onset, fALS, or with no molecular diagnosis at the time of inclusion were prioritized for NGS sequencing by referring clinicians. Most of the included patients had previously been analyzed with Sanger sequencing for *SOD1*, *C9orf72*, *TARDBP,* and *FUS* variants. Patients with definite, probable, laboratory supported, or possible ALS were included in the study, according to El Escorial revised criteria [82]. Blood samples were obtained for diagnostic purposes and stored in our tissue bank, after informed consent. Progression rate was defined by the slope on the revised ALS functional rating scale (ALSFRS-R). Seventeen patients (20.5%) had fALS. The mean age of onset of our cohort was 56.02 ± 13.9 years (range 19–81 years), which is slightly lower as compared with previous population-based studies [83]. Fifty-six patients (67.5%) were males and 27 (32.5%) were females. Sixty-eight patients (81.9%) had spinal onset ALS and 15 patients (18.1%) had bulbar-onset ALS. All patients were of Caucasian ethnicity (Table S1).

### 4.2. DNA Preparation

Genomic DNA was extracted from peripheral EDTA-treated blood samples using the NucleoSpin^®^ Blood L kit (Macherey-Nagel, Düren, Germany). The quality of genomic DNA was carefully evaluated and quantified by agarose gel electrophoresis, NanoDrop ND-1000 spectrophotometer (Thermo Fisher Scientific, Wilmington, DE) and Qubit 2.0 fluorometer using the Qubit dsDNA BR assay (Invitrogen, Merelbeke, Belgium). Only high-quality DNA samples (1.8–2.0 260/280 ratio and 2.0–2.2 260/230 ratio) were used for NGS analysis.

### 4.3. Targeted Next Generation Sequencing (NGS)

Genes for the ALS panel were selected from ALSoD (http://alsod.iop.kcl.ac.uk/) and from the Neuromuscular Disease Center (http://neuromuscular.wustl.edu/time/hmsn.html) databases. We selected major ALS genes previously associated with disease-causing mutations in patients with ALS. Moreover, we added to the panel a few non-ALS genes that we selected based on their association with hereditary motor syndromes, such as hereditary motor neuropathy (HMN), distal HMN or distal SMA (dHMN/dSMA), or axonal hereditary Charcot-Marie-Tooth neuropathy with predominant motor involvement (CMT2) (http://neuromuscular.wustl.edu/time/hmsn.html) [9]. In total, the following 28 genes divided in 2 categories were selected for the NGS targeted panel: ALS genes (*ALS2*, *ANG*, *DCTN1*, *DYNC1H1, ERBB4*, *FIG4*, *FUS*, *GARS*, *OPTN*, *PFN1*, *PLEKHG5*, *SETX*, *SOD1*, *SPAST*, *SPG7, SPG11*, *SQSTM1*, *TARDBP*, *TBK1*, *UBQLN2*, *VAPB*, and *VCP*) and other MND, HMN, and CMT2 genes (reported dSMA/hereditary motor (sensory) neuropathies (http://neuromuscular.wustl.edu/time/hmsn.html) (*BSCL2*, *HSPB1*, *HSPB3*, *HSPB8*, *MFN2*, and *TRPV4*) (Table S7).

The NGS targeted regions encompass 455 coding exons each including 20 intronic flanking bases and the 5′ and 3′ untranslated regions (UTRs). A customized design (TruSeq^®^ Custom Amplicon, TSCA; Illumina, San Diego, CA, USA) was employed with online probe design performed by the Design Studio (DS) software (https://designstudio.illumina.com/). The regional source of coding exons was extracted from the UCSC database (Genome Browser human GRCh37/hg19). A 98% coverage was predicted for the targeted region, 127,008 bp long, with a total of 968 amplicons. Targeted resequencing was performed using the MiSeq^®^ sequencing platform according to the manufacturer’s procedure (Illumina, San Diego, CA, USA). We analyzed single variants reported in the FASTQ and VCF output file with a commercially available NextGENe^®^ software (version V4.0.1 SoftGenetics^®^, State College, PA, USA) and visualized via Integrative Genome Viewer (IGV) software (http://www.broadinstitute.org/software/igv/) [84,85]. The *C9orf72* repeat expansion was analyzed in all the patients, using both amplicon-length and repeat-primed polymerase chain reactions, as described before [86].

### 4.4. Filters

All the regions with a sequencing depth <30 were considered to be not suitable for analysis, according to the guidelines of the American College of Medical Genetics and Genomics [77]. All variants with coverage depth >30 were filtered based on the following criteria [87]: (i) Qscore > 30; (ii) variants with minor allele frequencies (MAF) > 0.01 identified in the dbSNP150 database (www.ncbi.nlm.nih.gov/projects/SNP/) or Exome Aggregation Consortium Sequencing Project (ExAC; exac.broadinstitute.org) were filter out, in agreement with the ACMG classification criteria [77], with the exception of variants reported as pathogenic or of uncertain significance [8,18,73,87]; (iii) synonymous changes were excluded except for those located within or near splice sites. The impact of candidate variants was evaluated using prediction tools: Sorting Intolerant from Tolerant software (SIFT; sift.jcvi.org) [88], Polymorphism Phenotyping (PolyPhen-2; genetics.bwh.harvard.edu/pph2/) [89], and Mutation Taster (www.mutationtaster.org/) (Table S8). Clinical significance of reported variants was assessed on the basis of the ACMG guidelines [77]. A cohort of 332 non-neurological unrelated Italian patients, for whom NGS exome sequencing data were available from our in-house database, was selected as the control group. Variants in common between ALS patients and controls were excluded.

### 4.5. Sanger Sequencing Validation

All the identified variants were confirmed by Sanger sequencing. We designed primers using Primer3 (http://biotools.umassmed.edu/bioapps/primer3_www.cgi) to perform a direct sequencing. The PCR products were purified using AMPure (Agencourt-Beckmann Coulter, Inc., Brea, CA, USA), then, sequenced in both directions using a Big Dye Terminator v1.1 Cycle Sequencing Kit (Applied Biosystems Foster City, CA, USA). Sequencing products were purified using a Big Dye X-Terminator Kit (Applied Biosystems Foster City, CA, USA) and run on an ABI 3730 Genetic Analyzer (Applied Biosystems Foster City, CA, USA). Amplicon Sequences were compared with the reference sequence (hg19) using Sequencer 5.0 Software (Gene Codes). Primers are available on request. The validated variants were reported in the results depending on whether they were previously reported in the ALS literature, in the non-ALS literature, in population databases (including those reported only in dbSNP), or never reported (novel variants).

### 4.6. Statistical Analysis

Descriptive statistics are reported as count and percentage, for categorical variables, or mean and standard deviation, for continuous variables. The Chi-squared test, with Bonferroni correction for multiple comparisons, was used to explore differences in gene variant frequencies between ALS patients and non-neurological controls (Table S5). Kaplan–Meier univariate analyses were carried out to determine the effect of the burden of rare variants at MAF < 0.01 [8,18,73,87] on survival (defined as time from symptoms onset to death or tracheostomy), time needed to reach KING stage 4 (defined as the time from symptom onset to PEG or NIMV positioning), and age of onset. Moreover, we confirmed the results of survival analysis at the more stringent cut off of MAF < 0.001 [70]. Log-rank tests were used to test for differences between groups. Subsequently, multivariable analysis with Cox proportional hazards model (stepwise backward) was performed to estimate the proportional hazard ratio (HR) of rare variant burden on survival and on time needed to reach KING stage 4. Cox regressions were adjusted for the following factors known to influence survival in ALS patients: site of symptoms onset, diagnostic delay, presence of dementia, age at the onset, and progression rate, defined by the slope on the revised ALS functional rating scale (ALSFRS-R), i.e., ΔALSFRS-R = (48 minus ALSFRS-R score )/(date of the ALSFRS-R score minus date of onset) [77,89]. In the survival analysis, we treated all rare variants equally, and did not take into account whether some variants could be predicted to be more deleterious than others, according to previous ALS studies and since reliable models for rare variants interactions are still lacking [17,70,71]. Statistical analyses were performed using IBM Statistical Package for Social Science (SPSS) version 20, with *p*-value < 0.05.

## Figures and Tables

**Table 1:** Variants identified by next generation sequencing (NGS).

PT-ID ^a^	Gene Name	cDNA Change	Protein Change	dbSNP ID ^b^	ACMG Classification	Global MAF ^c^	Population MAF ^d^	SIFT Score	Polyphen Score	Mutation Taster Pred	CADD
**ALS literature**											
ALS_78	*ALS2*	c.1115C > G	p.Pro372Arg	rs190369242	3	0.00130	0.00220	0.64 (T)	0.919 (D)	0.683 (D)	19.65
ALS_6	*OPTN*	c.941A > T	p.Gln314Leu	rs142812715	3	0.00017	0.00030	0.01 (D)	0.999 (D)	0.993 (D)	27.70
ALS_56	*SETX*	c.654G > C	p.Lys218Asn	rs117861188	3	0.00033	0.00060	0.0 (D)	0.961 (D)	0.900 (D)	23.80
ALS_34	*SOD1*	c.203T > C	p.Leu68Pro	CM110553	5	0.00000	0.00000	0.21 (T)	0.001 (B)	0.99 (N)	6.15
ALS_6	*SOD1*	c.217G > A	p.Gly73Ser	rs121912455	5	0.00000	0.00000	0.0 (D)	0.970 (D)	0.999 (D)	29.30
ALS_23	*TBK1*	c.1190T > C	p.Ile397Thr	rs755069538	5	0.00010	0.00030	0.31 (T)	0.039 (B)	0.999 (D)	22.80
**ALS and non-ALS literature**											
ALS_5	*SETX*	c.59G > A	p.Arg20His	rs79740039	3	0.00683	0.01051	0.25 (T)	0.001 (B)	0.999 (N)	3.43
ALS_18	*SETX*	c.59G > A	p.Arg20His								
ALS_60	*SETX*	c.59G > A	p.Arg20His								
ALS_4	*SPG11*	c.1348A > G	p.Ile450Val	rs3759873	1	0.01672	0.00450	0.78 (T)	0 (B)	0.994 (D)	6.31
ALS_30	*SPG11*	c.3037A > G	p.Lys1013Glu	rs111347025	2	0.00861	0.01448	0.73 (T)	0.002 (B)	0.963 (D)	22.70
ALS_43	*SPG11*	c.3037A > G	p.Lys1013Glu								
ALS_32	*SPG11*	c.6224A > G	p.Asn2075Ser	rs140824939	3	0.00310	0.00489	0.49 (T)	0 (B)	0.999 (N)	0.28
**Non-ALS literature**											
ALS_2	*BSCL2*	c.844G > A	p.Ala282Thr(218)^§^	rs190842600	3	0.00022	0.00000	0.08 (T)	1 (D)	0.999 (D)	25.90
ALS_53	*BSCL2*	c.1033C > T	p.Arg345Trp(281)^§^	rs767820877	3	0.00000	0.00000	0.02(D)	0.987 (D)	0.999 (D)	21.40
ALS_41	*HSPB3*	c.347G > C	p.Arg116Pro	rs150931007	4	0.00011	0.00010	0 (D)	1 (D)	0.999 (D)	29.70
ALS_1	*MFN2*	c.1574A > G	p.Asn525Ser	rs145654854	3	0.00017	0.00021	1 (T)	0 (B)	0.753 (N)	19.62
ALS_6	*SETX*	c.4612C > T	p.Arg1538Trp	rs147018359	3	0.00000	0.00000	0.21 (T)	0 (B)	0.999 (N)	17.31
ALS_29	*SETX*	c.4612C > T	p.Arg1538Trp								
ALS_30	*SPG11*	c.5986_5987insT	p.Cys1996Leufs*4	rs312262775	5	0.00002	0.00000	-	-	-	-
ALS_7	*SQSTM1*	c.352C > T	p.Pro118Ser	rs200152247	3	0.00006	0.00010	0.54 (T)	0.009 (B)	0.999 (D)	20.90
ALS_34	*SQSTM1*	c.802C > G	p.Leu268Val	rs753685955	3	0.00001	0.00000	1 (T)	0.004 (B)	0.824 (N)	17.73
**Variants present only in population databases**											
ALS_64	*BSCL2*	c.689G > A	p.Ser230Asn (166) ^§^	rs778378228	4	0.00001	0.00000	0.11 (T)	0.820 (P)	0.824 (N)	23.40
ALS_67	*BSCL2*	c.785C > T	p.Ala262Val(198) ^§^	rs140896339	3	0.00028	0.00000	0.50 (T)	0.085(B)	0.999 (N)	18.85
ALS_7	*BSCL2*	c.1057T > A	p.Ser353Thr(189) ^§^	rs769769807	3	0.00001	0.00000	0.17 (T)	0.006 (B)	0.999 (N)	6.03
ALS_69	*BSCL2*	c.1282C > T	p.Pro428Ser(364) ^§^	rs369732238	3	0.00006	0.00010	0.1 (T)	0.573 (P)	0.905 (N)	18.59
ALS_43	*BSCL2*	c.1300T > C	p.Ser434Pro(370) ^§^	rs199584887	3	0.00006	0.00000	0.44 (T)	0.001 (B)	0.999 (N)	1.58
ALS_52	*DCTN1*	c.1486G > C	p.Val496Leu	rs773897036	4	0.00002	0.00000	0.06 (T)	0.984 (D)	0.999 (D)	23.50
ALS_13	*DYNC1H1*	c.265G > A	p.Gly89Ser	rs749973847	3	0.00002	0.00000	0.74 (T)	0.019 (B)	0.999 (D)	22.80
ALS_40	*DYNC1H1*	c.3748G > A	p.Val1250Met	rs369914512	3	0.00006	0.00010	0.07 (T)	0.978 (D)	0.999 (D)	29.20
ALS_41	*DYNC1H1*	c.12213C > T ^#^	p.Ile4071Ile	rs746950373	3	0.00002	0.00000	0.48 (T)	-	1 (D)	12.50
ALS_33	*HSPB1*	c.403T > G	p.Ser135Ala	rs766728475	4	0.00002	0.00000	0.07 (T)	0.623 (P)	0.999 (D)	25.40
ALS_53	*HSPB3*	c.199G > A	p.Gly67Ser	rs35258119	3	0.00727	0.00000	0.2 (T)	0.036 (B)	0.999 (N)	17.28
ALS_4	*HSPB3*	c.346C > T	p.Arg116X	rs757339596	4	0.00003	0.00000	-	-	0.999 (D)	11.58
ALS_58	*PLEKHG5*	c.3049G > A	p.Gly1017Arg	rs755699992	3	0.00000	0.00000	0.56 (T)	0.047 (B)	0.999 (N)	0.16
ALS_15	*SETX*	c.3182C > T	p.Pro1061Leu	rs12352982	3	0.01604	0.00000	1 (T)	0 (B)	0.999 (N)	0.90
ALS_46	*SETX*	c.7435A > G	p.Ile2479Val	rs536912256	3	0.00006	0.00000	0.43 (T)	0.001 (B)	0.999 (N)	0.76
ALS_73	*SPG11*	c.1675T > A	p.Ser559Thr	rs773680273	3	0.00001	0.00000	0.2 (T)	0.990 (D)	0.591 (D)	20.80
ALS_27	*SPG11*	c.2764G > A	p.Val922Ile	rs139399250	3	0,00006	0,00010	0.43 (T)	0.002(B)	0.999 (N)	2.25
ALS_67	*SPG11*	c.6201A > T ^#^	p.Gly2067Gly	rs764991726	3	0,00001	0,00000	1 (T)	-	0.987 (D)	7.48
**Novel candidate ALS-associated variants**											
ALS_34	*DYNC1H1*	c.4183A > C	p.Lys1395Gln	-	4	-	-	0.12 (T)	1.000 (D)	0.999 (D)	34.00
ALS_22	*DYNC1H1*	c.5303G > T	p.Ser1768Ile	-	3	-	-	0.1 (T)	0.028 (B)	0.999 (N)	15.71
ALS_27	*FIG4*	c.1030C > A	p.Pro344Thr	-	4	-	-	0.37 (T)	0.875 (P)	0.999 (D)	25.40
ALS_21	*FUS*	c.1168 + 7A > G ^#^	-	-	3	-	-	-	-	-	-
ALS_40	*SETX*	c.5852A > T	p.His1951Leu	-	4	-	-	0.13 (T)	0.961 (D)	0.974 (D)	25.80
ALS_70	*SPG11*	c.4826delT	p.Met1609Serfs*31	-	4	-	-	-	-	1 (D)	8.99

^a^ PT-ID, patient identification code; ^b^ dbSNP150; ^c^ Global MAF, global allele counts were calculated from all subjects in the ExAc database; ^d^ MAF population, population allele count refers to European Ancestry subjects from the ExAc database; ^§^, the variants present in *BSCL2* gene has been reported the positions in both isoform NM_001122955.3 and NM_001130702.2 in bracket; ^#^, variants predicted as splice site by in silico tools; ACMG, American College of Medical Genetics and Genomics; ACMG classification: 1 = benign, 2 = likely benign, 3 = uncertain significance, 4 = likely pathogenic, 5 = pathogenic; MAF, minor allele frequency; SIFT: T = tolerated and D = deleterious; Polyphen: B = benign, P = possibly damaging, D = damaging; Mutation Taster: D = disease causing, N = polymorphism, A = disease causing automatic; CADD, combined annotation dependent depletion; CADD scores, scaled CADD scores (Phred like) for scoring deleteriousness. Variants in common between ALS patients and controls were excluded (Table S4).

**Table 2:** List of patients with multiple variants of interest.

Pt ID ^a^	Family	Variant 1 (ACMG)	Gene Category	Variant 2 (ACMG)	Gene Category	Variant 3 (ACMG)	Gene Category
ALS_34	fALS	*SOD1* p.Leu68Pro (5)	ALS	*SQSTM1* p.Leu268Val (3)	ALS	*DYNC1H1* p.Lys1395Gln (4)	ALS
ALS_6	sALS	*SOD1* p.Gly73Ser (5)	ALS	*OPTN* p.Gln314Leu (3)	ALS	*SETX* p.Arg1538Trp (3)	ALS
ALS_40	fALS	*C9orf72* expansion (5)	ALS	*DYNC1H1* p.Val1250Met (3)	ALS	*SETX* p.His1951Leu (4)	ALS
ALS_52	fALS	*C9orf72* expansion (5)	ALS	*DCTN1* p.Val496Leu (4)	ALS		
ALS_22	sALS	*C9orf72* expansion (5)	ALS	*DYNC1H1* p.Ser1768Ile (3)	ALS		
ALS_27	sALS	*SPG11* p.Val922Ile (3)	ALS	*FIG4* p.Pro344Thr (4)	ALS		
ALS_30	sALS	*SPG11* p.Lys1013Glu (2)	ALS	*SPG11* p.Cys1996Leufs*4 (5)	ALS		
ALS_33	sALS	*C9orf72* expansion (5)	ALS	*HSPB1* p.Ser135Ala (4)	Other/HMN/CMT2		
ALS_41	sALS	*DYNC1H1* p.Ile4071Ile (3)	ALS	*HSPB3* p.Arg116Pro (4)	Other/HMN/CMT2		
ALS_4	sALS	*SPG11* p.Ile450Val (1)	ALS	*HSPB3* p.Arg116X (4)	Other/HMN/CMT2		
ALS_43	fALS	*SPG11* p.Lys1013Glu (2)	ALS	*BSCL2* p.Ser434Pro (3)	Other/HMN/CMT2		
ALS_67	sALS	*SPG11* p.Gly2067Gly (3)	ALS	*BSCL2* p.Ala262Val (3)	Other/HMN/CMT2		
ALS_7	sALS	*SQSTM1* p.Pro118Ser (3)	ALS	*BSCL2* p.Ser353Thr (3)	Other/HMN/CMT2		
ALS_53	sALS	*BSCL2* p.Arg345Trp (3)	Other/CMT2/dSMA	*HSPB3* p.Gly67Ser (3)	Other/CMT2/dSMA		

^a^ PT-ID, patient identification code. Key: American College of Medical Genetics and Genomics (ACMG) classification: 1 = benign, 2 = likely benign, 3 = uncertain significance, 4 = likely pathogenic, 5 = pathogenic. sALS, sporadic amyotrophic lateral sclerosis and fALS, familial amyotrophic lateral sclerosis.

**Table 3:** Cox proportional hazards regression multivariate analysis on survival and time to reach King stage 4 (MAF < 0.01).

	Survival		Time to King Stage 4	
Factor	HR (95% CI)	*p* Value	HR (95% CI)	*p* Value
*Rare variants (n)*		<0.001		0.023
0	1		1	
1	1.97 (0.98–3.97)	0.220	1.10 (0.56–2.19)	0.777
≥2	5.61 (2.38–13.22)	<0.001	3.09 (1.37–6.97)	0.007
*Site of symptoms onset*				
Spinal	1	0.013	1	0.042
Bulbar	2.64 (1.23–5.70)		2.32 (1.03–5.19)	
*ALSFRS-R decline (points/month)*				
≤0.60	1	0.003	1	0.036
>0.60	4.38 (1.64–11.69)		2.83 (1.07–7.49)	
*Diagnostic delay (months)*				
≤10	ns	ns	ns	Ns
>10				
*Dementia*				
No	ns	ns	ns	Ns
Yes				
*Age at the onset (years)*				
<60	ns	ns	1	0.016
>60			2.17 (1.15–4.08)	

Variables included in the model: age at onset (<60 and >60); presence of dementia (yes or no); site of symptoms onset (bulbar and spinal); ALSFRS-R decline (≤0.60 and >0.60 points/months); diagnostic delay (≤10 months and >10 months); MAF (0, 1, and ≥2 variants). ALSFRS-R, ALS functional rating scale revised; MAF, minor allele frequency; HR, hazard ratio; CI, confidence interval; ns, not significant.

## Supplementary Materials

Supplementary materials can be found at https://www.mdpi.com/1422-0067/21/9/3346/s1. Table S1. Clinical and demographic characteristics of ALS patients. Table S2. Rare variants identified in this study and clinical features of the index patients. Table S3. Rare variants identified in non-neurological controls (664 Chromosomes). Table S4. Variants identified in this study and presence in controls. Table S5. Overview of variants identified in ALS patients and controls. Table S6. Cox proportional hazards regression multivariate analysis on survival and time to King stage 4 (MAF < 0.001). Table S7. Genes present in the panel. Table S8. Criteria for evaluation and filtering of the identified variants in this study. Figure S1. Kaplan-Meier univariate analysis for Survival (A) and time to King’s stage 4 (B) (MAF < 0.001). Black line: patients harboring no variants; green line: one rare variant; red line: two or more rare variants.

## Abbreviations

ALS	Amyotrophic lateral sclerosis
MND	Motor neuron diseases
UMN	Upper motor neurons
LMN	Lower motor neurons
fALS	Familial amyotrophic lateral sclerosis
sALS	Sporadic smyotrophic lateral sclerosis
SOD1	Superoxide Dismutase 1
C9orf72	Chromosome 9 open reading frame 72
TARDBP	TAR DNA binding protein
FUS	FUS RNA binding protein
CMT	Charcot-Marie-Tooth neuropathy
dHMN	Distal hereditary motor neuropathy
NGS	Next generation sequencing
ALS2	Alsin Rho guanine nucleotide exchange factor
ANG	Angiogenin
DCTN1	Dynactin subunit 1
ERBB4	Erb-B2 receptor tyrosine kinase 4
FIG4	FIG4 phosphoinositide 5-phosphatase
OPTN	Optineurin
PFN1	Profilin 1
SETX	Senataxin
SPAST	Spastin
SPG11	Spatacsin vesicle trafficking associated
SQSTM1	Sequestosome 1
UBQLN2	Ubiquilin 2
VAPB	VAMP associated protein B and C
VCP	Valosin containing protein
DYNC1H1	Dynein cytoplasmic 1 heavy chain 1
GARS	Glycyl-tRNA synthetase
PLEKHG5	Pleckstrin homology and RhoGEF domain containing G5
SPG7	Paraplegin matrix AAA peptidase subunit
BSCL2	Seipin lipid droplet biogenesis associated
HSPB1	Heat shock protein family B (Small) member 1
HSPB3	Heat shock protein family B (Small) member 3
HSPB8	Heat shock protein family B (Small) member 8
MFN2	Mitofusin 2
TRPV4	Transient receptor potential cation channel subfamily V member 4
TBK1	TANK-binding kinase 1
HSP	Hereditary spastic paraplegia
dSMA	Distal spinal muscular atrophy
POAG	Primary open angle glaucoma
AOA2	Ataxia with oculomotor apraxia type 2
PEO	Progressive external ophthalmoplegia
AD	Alzheimer disease
FTD	Frontotemporal dementia
SMA-LED	Spinal muscular atrophy with predominance of lower extremity involvement
PEG	Percutaneous endoscopic gastrostomy
NIMV	Non-invasive mechanical ventilation
