# Supplementary material for: Burden of Rare Variants in ALS and Axonal Hereditary Neuropathy Genes Influence Survival in ALS: Insights from a Next Generation Sequencing Study of an Italian ALS Cohort
Source: Int J Mol Sci. 2020 May 8;21(9):3346. doi: 10.3390/ijms21093346 (PMC7246633; doi:10.3390/ijms21093346)
Supplement: Supplementary file 1 [file ijms-21-03346-s001.pdf]

Table S1. Clinical and demographic characteristics of ALS patients

PT-ID[a]	Sex	Clinical Phenotype	Onset: Spinal (0) Bulbar (1)	Diagnostic delay (months)	Survival (months)	Onset to PEG (months)	Onset to NIMV (months)	Age at onset (Years)	Family History	FTD
ALS_1	F	Bulbar	1	4	16	-	-	73.8	sALS	NO
ALS_2	M	Classic	0	12	34	-	-	74.6	sALS	YES
ALS_3	M	Pyramidal	0	16	94	-	-	62.0	sALS	NO
ALS_4	M	Classic	0	10	33	-	-	74.2	sALS	NO
ALS_5	M	Classic	0	5	44	21	7	67.9	sALS	NO
ALS_6	M	Classic	0	11	26	26	19	58.6	sALS	NO
ALS_7	M	Classic	0	15	18	-	-	75.5	sALS	NO
ALS_8	F	Pyramidal	0	8	37	-	-	60.1	fALS	NO
ALS_9	M	Classic	0	6	10	-	8	73.1	sALS	NO
ALS_10	M	PLMN	0	24	72	-	-	43.0	sALS	NO
ALS_11	M	Classic	0	4	32	10	6	68.8	sALS	NO
ALS_12	M	Respiratory	0	3	72	-	5	68.2	sALS	NO
ALS_13	F	Bulbar	1	5	36	10	26	52.3	sALS	NO
ALS_14	M	PLMN	0	27	63	-	45	60.9	sALS	NO
ALS_15	F	Bulbar	1	4	43	28	-	46.3	sALS	NO
ALS_16	F	Pyramidal	0	7	27	19	12	65.2	sALS	NO
ALS_17	M	Pyramidal	0	3	43	20	14	59.9	sALS	NO
ALS_18	M	Pyramidal	0	16	60	49	40	66.0	sALS	NO
ALS_19	F	Classic	0	12	31	26	13	71.8	sALS	NO
ALS_20	M	Pyramidal	0	7	36	19	19	64.1	sALS	NO
ALS_21	M	Pyramidal	0	3	42	-	-	68.3	sALS	NO
ALS_22	M	Classic	0	9	32	16	28	60.5	sALS	NO
ALS_23	M	Pyramidal	0	18	71	-	48	65.0	sALS	NO
ALS_24	M	Pyramidal	0	2	14	-	-	60.3	sALS	NO
ALS_25	M	Pyramidal	0	21	78	60	-	54.8	sALS	NO
ALS_26	M	PUMN	0	3	83	-	-	37.4	sALS	NO
ALS_27	M	Flail Arms	0	20	48	-	25	56.8	sALS	NO
ALS_28	M	PLMN	0	3	11	11	10	61.6	fALS	NO
ALS_29	M	PLMN	0	23	76	-	-	55.4	sALS	NO
ALS_30	F	Classic	0	11	44	-	39	81.8	sALS	NO

ALS_31	F	Classic	0	94	190	-	-	21.3	sALS	NO
ALS_32	M	PLMN	0	51	226	-	-	46.5	sALS	NO
ALS_33	F	Flail Leg	0	4	29	-	-	52.4	sALS	NO
ALS_34	M	Classic	0	5	11	11	8	54.4	fALS	NO
ALS_35	F	Bulbar	1	4	21	-	18	71.2	sALS	NO
ALS_36	M	PLMN	0	36	102	-	-	61.5	sALS	NO
ALS_37	F	Bulbar	1	3	67	34	34	67.8	sALS	NO
ALS_38	M	PUMN	0	13	75	-	60	65.2	sALS	NO
ALS_39	M	PUMN	0	16	71	-	-	59.8	sALS	NO
ALS_40	M	Pyramidal	0	1	14	7	7	56.6	fALS	NO
ALS_41	F	Classic	0	-	-	-	-	-	sALS	YES
ALS_42	M	Bulbar	0	34	125	-	-	60.8	sALS	NO
ALS_43	M	Bulbar	1	12	30	-	-	58.9	fALS	NO
ALS_44	M	Bulbar	1	37	50	-	50	62.4	sALS	NO
ALS_45	M	Bulbar	1	1	14	12	12	61.6	sALS	NO
ALS_46	M	Flail Leg	0	21	31	41	29	76.6	sALS	NO
ALS_47	F	Bulbar	1	8	44	24	-	62.5	sALS	NO
ALS_48	F	PLMN	0	94	154	-	-	31.6	sALS	NO
ALS_49	F	Pyramidal	0	6	31	-	-	42.0	sALS	NO
ALS_50	M	PLMN	0	95	201	-	-	8.8	sALS	NO
ALS_51	M	Classic	0	8	47	-	-	27.2	sALS	NO
ALS_52	M	Classic	0	6	28	-	-	44.4	fALS	NO
ALS_53	M	Pyramidal	0	3	17	-	-	35.8	sALS	NO
ALS_54	M	Pyramidal	0	10	69	34	41	38.6	sALS	NO
ALS_55	F	Bulbar	1	11	31	-	-	69.4	fALS	NO
ALS_56	M	PUMN	0	206	305	-	-	55.3	fALS	NO
ALS_57	M	Classic	0	5	22	-	-	70.4	fALS	NO
ALS_58	M	Pyramidal	0	6	60	-	-	33.6	sALS	NO
ALS_59	F	Pyramidal	0	59	59	-	67	35.8	sALS	NO
ALS_60	F	PUMN	0	42	157	-	-	32.6	sALS	NO
ALS_61	M	PLMN	0	58	246	-	-	22.3	sALS	NO
ALS_62	F	Pyramidal	0	55	73	-	-	40.8	sALS	NO
ALS_63	M	Classic	0	3	31	-	-	43.3	sALS	NO
ALS_64	F	Bulbar	1	12	55	-	-	44.9	sALS	NO
ALS_65	M	Bulbar	1	14	41	27	-	58.0	fALS	NO

ALS_66	F	Bulbar	1	4	11	12	10	77.0	fALS	NO
ALS_67	M	Flail Arms	0	54	165	-	60	57.0	sALS	NO
ALS_68	M	Bulbar	1	12	146	-	-	53.0	fALS	NO
ALS_69	M	Classic	0	16	27	-	20	58.0	fALS	NO
ALS_70	M	Flail Arms	0	138	212	-	-	60.0	sALS	YES
ALS_71	M	Classic	0	34	110	73	65	69.0	sALS	NO
ALS_72	M	PUMN	0	2	98	-	-	56.0	fALS	NO
ALS_73	F	Pyramidal	0	17	17	-	-	46.0	sALS	NO
ALS_74	F	Classic	0	5	52	31	22	45.0	sALS	NO
ALS_75	M	Classic	0	4	31	28	22	52.0	sALS	NO
ALS_76	M	PUMN	0	10	107	-	77	59.0	sALS	NO
ALS_77	F	Classic	0	7	22	-	12	62.0	fALS	NO
ALS_78	F	Classic	0	6	25	19	14	73.0	sALS	NO
ALS_79	F	-	-	-	87	-	-	52.0	sALS	NO
ALS_80	M	Respiratory	0	6	71	31	13	71.0	fALS	NO
ALS_81	F	Pyramidal	0	9	35	-	-	55.0	fALS	YES
ALS_82	M	Flail Arms	0	22	63	-	28	68.0	sALS	NO
ALS_83	M	Classic	0	7	19	-	-	55.0	sALS	NO

[a] PT-ID, patient identification code. Key: FTD = Frontotemporal dementia; PLMN = predominantly lower motor neuron disease; PUMN = predominantly upper motor neuron disease; sALS = sporadic amyotrophic lateral sclerosis; fALS = familial amyotrophic lateral sclerosis; NIMV: Non-invasive Mechanical Ventilation

Table S2. Rare variants identified in this study and clinical features of the index patients

Pt-ID[a]	Gene Variants	Sex	Age of Onset (years)	Family History	Survival (months)	ALSFRS-r at Diagnosis	Site of Onset	Clinical Phenotype	Presence FTD
ALS_78	ALS2 p.Pro372Arg	F	73.0	sALS	25	3	Spinal Upper	Classic	NO
ALS_64	BSCL2 p.Ser230Asn	F	44.9	sALS	44	46	Bulbar	Bulbar	NO
ALS_67	BSCL2 p.Ala262Val, SPG7 p.Ala510Val, SPG11 p.Gly2067Gly	M	57.0	sALS	161	21	Spinal Upper	Flail Arms	NO
ALS_2	BSCL2 p.Ala282Thr	M	74.6	sALS	34	44	Spinal Upper	Classic	YES
ALS_53	BSCL2 p.Arg345Trp, HSPB3 p.Gly67Ser	M	35.8	sALS	17	36	Spinal Upper	Pyramidal	NO
ALS_7	BSCL2 p.Ser353Thr, SQSTM1 p.Pro118Ser	M	75.5	sALS	19	42	Spinal Upper	Classic	NO
ALS_69	BSCL2 p.Pro428Ser	M	58.0	fALS	27	16	Spinal Upper	Classic	NO
ALS_43	BSCL2 p.Ser434Pro, SPG11 p.Lys1013Glu	M	58.9	fALS	30	-	Bulbar	Bulbar	YES
ALS_8	C9orf72 Expansion	F	60.1	fALS	37	26	Spinal Lower	Pyramidal	YES
ALS_55	C9orf72 expansion	F	69.4	fALS	31	-	Bulbar	Bulbar	NO
ALS_42	C9orf72 expansion	M	60.8	sALS	114	42	-	Kennedy	NO
ALS_52	C9orf72 expansion, DCTN1p.Val496Leu	M	44.4	fALS	28	-	Spinal Upper	Classic	NO

ALS_22	C9orf72 expansion, DYNC1H1p.Ser1768Ile	M	60.5	sALS	32	27	Spinal Lower	Classic	NO
ALS_40	C9orf72 expansion, DYNC1H1 p.Val1250Met, SETX p.His1951Leu	M	56.6	fALS	14	28	Spinal Upper	Pyramidal	NO
ALS_33	C9orf72 expansion, HSPB1 p.Ser135Ala	F	52.4	sALS	29	31	Bulbar	Flail Legs	NO
ALS_52	DCTN1 p.Val496Leu, C9orf72 expansion	M	44.4	fALS	28	-	Spinal Upper	Classic	NO
ALS_13	DYNC1H1 p.Gly89Ser	F	52.3	sALS	36	41	Bulbar	Bulbar	NO
ALS_40	DYNC1H1 p.Val1250Met, C9orf72 expansion, SETX p.His1951Leu	M	56.6	fALS	14	28	Spinal Upper	Pyramidal	NO
ALS_34	DYNC1H1 p.Lys1395Gln, SOD1 p.Leu67Pro, SQSTM1 p.Leu268Val	M	54.4	fALS	11	-	Spinal Lower	Classic	NO
ALS_22	DYNC1H1 p.Ser1768Ile, C9orf72 expansion	M	60.5	sALS	32	27	Spinal Lower	Classic	NO
ALS_41	DYNC1H1 p.Ile4071Ile, HSPB3p.Arg116Pro	F	77.6	sALS	30	31	Spinal Lower	-	NO
ALS_27	FIG4 p.Pro344THr, SPG11 p.Val922Ile	M	56.8	sALS	48	45	Spinal Upper	Flail Arms	NO
ALS_21	FUS c.1168+7A>G	M	68.3	sALS	42	36	Spinal Lower	Pyramidal	NO
ALS_33	HSPB1 p.Ser135Ala, C9orf72 expansion	F	52.4	sALS	29	31	Bulbar	Flail Legs	NO
ALS_53	HSPB3 p.Gly67Ser, BSCL2 p.Arg345Trp	M	35.8	sALS	17	36	Spinal Upper	Pyramidal	NO
ALS_41	HSPB3 p.Arg116Pro, DYNC1H1 p.Ile4071Ile	F	77.6	sALS	30	31	Spinal Lower	-	NO

ALS_4	HSPB3 p.Arg116X, SPG11 p.Ile450Val	M	74.2	sALS	33	-	Spinal Lower	Classic	NO
ALS_1	MFN2 p.Asn525Ser	F	73.8	sALS	16	39	Bulbar	Bulbar	NO
ALS_6	OPTN p.Gln314Leu, SOD1 p.Gly73Ser, SETX p.Arg1538Trp	M	58.6	sALS	26	-	Spinal Upper	Classic	NO
ALS_58	PLEKHG5 p.Gly1017Arg	M	33.6	sALS	6	-	Spinal Lower	Pyramidal	NO
ALS_5	SETX p.Arg20His	M	67.9	sALS	44	32	Spinal Upper	Classic	NO
ALS_60	SETX p.Arg20His	F	32.6	sALS	155	-	Spinal Lower	PUMN	NO
ALS_18	SETX p.Arg20His	M	66.0	sALS	60	45	Spinal Lower	Pyramidal	NO
ALS_56	SETX p.Lys218Asn	M	55.3	fALS	294	36	Spinal Lower	PUMN	NO
ALS_15	SETX p.Pro1061Leu	F	46.3	sALS	43	44	Spinal Upper	Bulbar	YES
ALS_15	SETX p.Pro1061Leu	F	46.3	sALS	43	44	Spinal Upper	Bulbar	YES
ALS_29	SETX p.Arg1538Trp	M	55.4	sALS	76	43	Spinal Lower	PLMN	NO
ALS_6	SETX p.Arg1538Trp, SOD1 p.Gly73Ser, OPTN p.Gln314Leu	M	58.6	sALS	26	-	Spinal Upper	Classic	NO
ALS_40	SETX p.His1951Leu, C9orf72 expansion, DYNC1H1 p.Val1250Met	M	56.6	fALS	14	28	Spinal Upper	Pyramidal	NO
ALS_46	SETX p.Ile2479Val	M	76.6	sALS	31	27	Spinal Lower	Flail Legs	NO

ALS_34	SOD1 p.Leu68Pro, SQSTM1 p.Leu268Val, DYNC1H1 p.Lys1395Gln	M	54.4	fALS	11	-	Spinal Lower	Classic	NO
ALS_6	SOD1 p.Gly73Ser, OPTN p.Gln314Leu, SETX p.Arg1538Trp	M	58.6	sALS	26	-	Spinal Upper	Classic	NO
ALS_4	SPG11 p.Ile450Val, HSPB3 p.Arg116X	M	74.2	sALS	33	-	Spinal Lower	Classic	NO
ALS_73	SPG11 p.Ser559Thr	F	46.0	sALS	20	41	-	Pyramidal	NO
ALS_27	SPG11 p.Val922Ile, FIG4 p.Pro344Thr	M	56.8	sALS	48	45	Spinal Upper	Flail Arms	NO
ALS_43	SPG11 p.Lys1013Glu, BSCL2 p.Ser434Pro	M	58.9	fALS	30	-	Bulbar	Bulbar	YES
ALS_30	SPG11p.Lys1013Glu, SPG11 p.Cys1996Leufs*4	F	81.8	sALS	44	26	Spinal Upper	Classic	NO
ALS_70	SPG11 p.Met1609Serfs*31	M	60.0	sALS	212	40	Spinal Upper	Flail Arms	YES
ALS_30	SPG11p.Cys1996Leufs*4, SPG11 p.Lys1013Glu	F	81.8	sALS	44	26	Spinal Upper	Classic	NO
ALS_67	SPG11 p.Gly2067Gly, BSCL2 p.Ala262Val	M	57.0	sALS	161	21	Spinal Upper	Flail Arms	NO
ALS_32	SPG11 p.Asn2075Ser	M	46.5	sALS	115	44	Spinal Lower	PLMN	NO
ALS_7	SQSTM1 p.Pro118Ser, BSCL2 p.Ser353Thr	M	75.5	sALS	18	42	Spinal Upper	Classic	NO
ALS_34	SQSTM1 p.Leu268Val, SOD1 p.Leu68Pro, DYNC1H1 p.Lys1395Gln	M	54.4	fALS	11	-	Spinal Lower	Classic	NO
ALS_23	TBK1 p.Ile397Thr	M	65.0	sALS	71	39	Spinal Lower	Pyramidal	NO

[a] PT-ID, patient identification code. Key: sALS = sporadic amyotrophic lateral sclerosis; fALS = familial amyotrophic lateral sclerosis; PLMN = predominantly lower motor neuron disease; PUMN = predominantly upper motor neuron disease

Table S3. Rare variants identified in non-neurological controls (664 Chromosomes).

Nr Subjects[a]	Gene Name	cDNA Change	Protein Change	dbSNP ID[b]	Global MAF[c]	Population MAF[d]	SIFT Score	Polyphen Score	CADD
1	*ALS2*	c.4948C>T	p.Gln1650Ter	-	-	-	-	-	-
1	*ALS2*	c.4886A>G	p.Asp1629Gly	rs761983607	0.00003	0.0000	0 (D)	0.999 (D)	32
1	*ALS2*	c.4281G>T	p.Arg1427Ser	rs746790680	0.00001	0.0000	0.01 (D)	0.96 (D)	26.3
1	*ALS2*	c.4119A>G	p.Ile1373Met §	rs61757691	0.00305	0.0048	0.04 (D)	0.109 (B)	21.9
1	*ALS2*	c.3860_3865delTGCCAG	p.Val1287_Pro1288del	rs759145161	0.00003	0.0000	-	-	-
1	*ALS2*	c.3814G>A	p.Asp1272Asn	rs200697299	0.00008	0.0001	0.12 (T)	0.056 (B)	23.3
1	*ALS2*	c.3440C>G	p.Ser1147Cys	rs777004596	0.00002	0.0000	0.02 (D)	0.412 (B)	32
1	*ALS2*	c.3410T>G	p.Leu1137Arg	rs750414152	0.00001	0.0000	0(D)	0.88 (P)	31
1	*ALS2*	c.3206G>A	p.Gly1069Glu §	rs200706696	0.00051	0.0008	0(D)	0.977 (D)	27
1	*ALS2*	c.2849C>T	p.Thr950Met	-	-	-	0(D)	0.608 (P)	-
1	*ALS2*	c.2389G>A	p.Gly797Arg	-	-	-	0(D)	0.999 (D)	-
1	*ALS2*	c.2171-2A>G	-	-	-	-	-	-	-
1	*ALS2*	c.853A>G	p.Arg285Gly	rs778865969	0.00002	0.0000	0.41 (T)	0 (B)	12.88
1	*BSCL2*	c.1369C>T	p.Pro457Ser	rs769048111	0.00001	0.0000	0.88 (T)	0.002 (B)	15.88
8	*BSCL2*	c.1280T>C	p.Leu427Pro §	rs145649423	0.00392	0.0056	0.07 (T)	0.081 (B)	21.2
1	*BSCL2*	c.1193C>G	p.Pro398Arg	rs778931376	0.00001	0.0000	0.08 (T)	0.361(B)	25.6
1	*BSCL2*	c.986G>T	p.Arg329Leu	-	-	-	0.03 (D)	0.689 (P)	-
1	*BSCL2*	c.845C>T	p.Ala282Val	rs185341934	0.00165	0.0001	0 (D)	1 (D)	27.5
1	*BSCL2*	c.359A>G	p.Tyr120Cys	rs370905417	0.00011	0.0002	0 (D)	1 (D)	29.6
1	*BSCL2*	c.-4G>A	-	-	-	-	-	-	-
1	*DCTN1*	c.3803A>G	p.Gln1268Arg	rs751431467	0.00001	0.0000	0.01 (D)	0.507 (P)	27
6	*DCTN1*	c.3529+5G>A	-	rs72466494	0.00590	0.0082	-	-	19.51
1	*DCTN1*	c.2969G>A	p.Arg990His	rs369129714	0.00000	0.00000	0.01 (D)	0.95 (D)	28.9
1	*DCTN1*	c.2432C>G	p.Pro811Arg	rs150928856	0.00000	0.00000	0.02 (D)	0.536 (P)	25.2
1	*DCTN1*	c.2429C>T	p.Ala810Val	rs372500630	0.00001	0.00000	0.29 (T)	0.034 (B)	24.9

1	*DCTN1*	c.1595G>A	p.Arg532Gln	rs759306485	0.00001	0.00000	0.14 (T)	0.354 (B)	28.1
1	*DCTN1*	c.1267A>G	p.Ile423Val	rs766531179	0.00001	0.00000	0.64 (T)	0.011 (B)	21.1
1	*DCTN1*	c.999C>G	p.Asp333Glu	rs200952455	0.00000	0.00000	1 (T)	0.004 (B)	0.001
1	*DCTN1*	c.596C>A	p.Pro199Gln	-	-	-	0.07 (D)	0.974 (P)	-
2	*DYNC1H1*	c.173A>C	p.Glu58Ala	-	-	-	0.75 (T)	0.006 (B)	-
1	*DYNC1H1*	c.2098G>A	p.Asp700Asn	-	-	-	0.18 (T)	0.049 (B)	-
1	*DYNC1H1*	c.5009A>G	p.Asn1670Ser	rs758191823	0.00001	0.00001	0.8 (T)	0.021 (B)	16.96
1	*DYNC1H1*	c.7203A>C	p.Lys2401Asn	rs150888094	0.00032	0.0004	0.5 (T)	0.028 (B)	11.89
1	*DYNC1H1*	c.8303C>T	p.Pro2768Leu	-	-	-	0.01 (D)	0.93 (D)	-
1	*DYNC1H1*	c.10522C>A	p.Leu3508Ile	rs149496322	0.00014	0.0002	0.01 (D)	0.919 (D)	26
1	*DYNC1H1*	c.11806G>T	p.Val3936Leu	-	-	-	0.77 (T)	0.003 (B)	-
1	*DYNC1H1*	c.11942C>G	p.Thr3981Arg	rs138428684	0.00341	0.0053	0.08 (T)	0.051 (B)	26.7
1	*DYNC1H1*	c.12047C>G	p.Ser4016Cys	rs370026529	0.00002	0.00000	0.02 (D)	0.563 (P)	23.4
1	*DYNC1H1*	c.12259G>T	p.Ala4087Ser	-	-	-	0.03 (D)	0.858 (P)	-
1	*ERBB4*	c.2845G>A	p.Val949Ile	rs376298364	0.00005	0.00000	0.04 (D)	0.167 (B)	23.4
1	*ERBB4*	c.2379G>T	p.Gln793His	rs142841164	0.00000	0.00000	0 (D)	0.996 (D)	24.2
1	*ERBB4*	c.1395C>A	p.Asn465Lys	rs200755699	0.00008	0.0001	0.25 (T)	0.125 (B)	17.29
5	*ERBB4*	c.1122T>G	p.His374Gln §	rs76603692	0.00250	0.0030	0.34 (T)	0.046 (B)	17.24
1	*ERBB4*	c.1034C>T	p.Ala345Val	-	-	-	0.64 (T)	0.036 (B)	-
8	*ERBB4*	c.882A>G	p.Pro294Pro §	rs77309171	0.00316	0.00531	1 (T)	-	13.50
2	*FIG4*	c.122T>C	p.Ile41Thr §	rs121908287	0.00099	0.0014	0 (D)	0.994 (D)	25.8
1	*FIG4*	c.2324G>A	p.Arg775His	rs775280287	0.00002	0.00000	0.07 (T)	0.009 (B)	26.5
1	*FIG4*	c.2713_*26delCGCTACCTG TGAAAAGAGCGCAGGTC CACCTGGTGGAC	-	-	-	-	-	-	-
1	*FUS*	c.238G>A	p.Gly80Ser	rs776474571	0.00006	0.0001	0.09 (T)	0.597 (P)	21.8
1	*FUS*	c.620G>A	p.Gly207Asp	-	-	-	0.36 (T)	0.030 (B)	-
3	*FUS*	c.684_686dupCGG	p.Gly231dup	rs72550890	0.005	0.00000	-	-	-

1	*FUS*	c.681_689delCGGCGGTGG	p.Gly229_Gly231del §	rs767564995	0.0001	0.0002	-	-	-
1	*FUS*	c.1348C>T	p.Pro450Ser	rs201533156	0.00014	0.0001	0 (D)	0.96 (D)	25.4
1	*FUS*	c.1396G>A	p.Gly466Ser	rs747547178	0.00003	0.00000	0.26 (T)	0.934 (D)	23.5
1	*HSPB1*	c.11G>A	p.Arg4His	rs748730374	0.00003	0.0000	0.04 (D)	0.999 (D)	34
1	*HSPB1*	c.16G>A	p.Val6Ile	rs1049324	0.00015	0.0002	0.52 (T)	0.083 (B)	17.12
3	*HSPB1*	c.178C>T	p.Pro60Ser	rs61751217	0.0029	0.0055	0.24 (T)	0.001 (B)	10.22
1	*HSPB3*	c.397C>T	p.Leu133Phe	-	-	-	0.56 (T)	0.014 (B)	-
1	*MFN2*	c.464G>A	p.Arg155Lys	-	-	-	1 (T)	0.002 (B)	-
1	*MFN2*	c.816+5G>A	-	rs770949123	0.00001	0.00000	-	-	21.6
1	*MFN2*	c.892G>A	p.Gly298Arg	rs41278630	0.00206	0.0030	0.43 (T)	0.358 (B)	19.35
1	*MFN2*	c.1036G>T	p.Glu346Ter	-	-	-	-	-	-
1	*MFN2*	c.1117C>G	p.Arg373Gly	-	-	-	0.01 (D)	0.992 (D)	-
1	*MFN2*	c.1151G>A	p.Arg384Gln	rs565042936	0.00001	0.00000	0.66 (T)	0 (B)	19.97
1	*MFN2*	c.1253G>A	p.Arg418Gln	rs766998571	0.00001	0.00000	0.02 (D)	0.032 (B)	25.1
2	*MFN2*	c.1403G>A	p.Arg468His §	rs138382758	0.00229	0.0033	0.1 (T)	0.466 (P)	23.3
2	*MFN2*	c.2113G>A	p.Val705Ile	rs142271930	0.00635	0.0098	0.19 (T)	0.041 (B)	15.75
1	*OPTN*	c.46C>G	p.Pro16Ala	rs758942502	0.00002	0.00000	0.17 (T)	0.237 (B)	9.15
1	*OPTN*	c.160C>A	p.Leu54Met	-	-	-	0 (D)	1 (D)	-
1	*OPTN*	c.644G>A	p.Arg215Lys	rs369585614	0.00001	0.00000	0.14 (T)	0.04 (B)	24.5
1	*OPTN*	c.909C>A	p.Asn303Lys	rs200114679	0.00065	0.0002	0.73 (T)	0.008 (B)	0.987
1	*OPTN*	c.1634G>A	p.Arg545Gln	rs75654767	0.00290	0.0004	1 (T)	0.004 (B)	0.053
1	*PLEKHG5*	c.3145C>T	p.Pro1049Ser	rs373021205	0.00000	0.00000	0.13 (T)	0.986 (D)	14.60
1	*PLEKHG5*	c.2983A>C	p.Thr995Pro	rs187886272	0.00000	0.00000	0.19 (T)	0.019 (B)	14.33
1	*PLEKHG5*	c.2695G>A	p.Gly899Ser	rs202191898	0.00000	0.00000	0 (D)	0.972 (D)	27.8
15	*PLEKHG5*	c.2400_2405delGGAGGA	p.Glu801_Glu802del	rs113541584	0.097	0.065	-	-	-
2	*PLEKHG5*	c.2403_2405delGGA	p.Glu802del	-	-	-	-	-	-
1	*PLEKHG5*	c.1966G>A	p.Ala656Thr	rs143545780	0.00000	0.00000	0.89 (T)	0.17 (B)	15.09
1	*PLEKHG5*	c.1917+2T>G	-	-	-	-	-	-	-

1	*PLEKHG5*	c.1234C>T	p.Arg412Trp	rs148232621	0.00000	0.00000	0 (D)	0.952 (D)	23.7
2	*PLEKHG5*	c.1231C>T	p.Arg411Trp	rs140202670	0.0011	0.0007	0.01 (D)	0.968 (D)	23.7
1	*PLEKHG5*	c.1165G>A	p.Asp389Asn	rs61730399	0.00262	0.0040	0.08 (T)	0.763 (P)	18.99
1	*PLEKHG5*	c.1094_1114delCCAGGCTG CCCCGGGGGCTGC	p.Pro365_Leu371del	-	-	-	-	-	-
1	*PLEKHG5*	c.1004C>T	p.Ser335Phe	rs376782083	0.0001	0.0002	0 (D)	0.989 (D)	27.7
2	*PLEKHG5*	c.956A>G	p.Asp319Gly	rs199794578	0.0005	0.0010	0.06 (T)	0.328 (B)	25.1
1	*PLEKHG5*	c.928G>A	p.Gly310Ser	rs146651455	0.0015	0.00000	0.61 (T)	0.036 (B)	2.676
1	*PLEKHG5*	c.778G>A	p.Ala260Thr	rs527341275	0.00000	0.00000	0.23 (T)	0.002 (B)	1.299
1	*PLEKHG5*	c.677-2A>G	-	rs144750655	0.00020	0.0003	-	-	7.581
1	*PLEKHG5*	c.676G>T	p.Ala226Ser	-	-	-	0.01 (D)	0.006 (B)	-
1	*PLEKHG5*	c.668G>A	p.Arg223His	rs150152888	0.00005	0.00000	0.08 (T)	0.607 (P)	21.2
1	*PLEKHG5*	c.544G>A	p.Val182Met	rs141032388	0.00195	0.0028	0 (D)	0.997 (P)	26.2
1	*PLEKHG5*	c.326G>A	p.Arg109His	rs199745947	0.00000	0.00000	0.01 (D)	0.629 (P)	25.9
1	*PLEKHG5*	c.319C>T	p.Arg107Cys §	rs111400494	0.00283	0.00479	0.01 (D)	0.049 (P)	24.6
1	*PLEKHG5*	c.301G>A	p.Val101Met	rs112530241	0.00062	0.00000	0.1 (T)	0.931 (D)	22.7
1	*PLEKHG5*	c.118C>T	p.Pro40Ser	rs201669114	0.00143	0.0022	0.02 (D)	0.986 (D)	24.9
4	*SETX*	c.7640T>C	p.Ile2547Thr	rs151117904	0.00342	0.0044	0.49 (T)	0.001 (B)	2.619
1	*SETX*	c.5591A>C	p.Gln1864Pro	rs375747001	0.00003	0.0001	0 (D)	0.836 (P)	24.9
2	*SETX*	c.5051C>G	p.Ser1684Cys	rs140116005	0.00012	0.0001	0.07 (T)	0.592 (P)	16.85
1	*SETX*	c.4982C>G	p.Pro1661Arg	rs146873848	0.00031	0.0002	0.03 (D)	0 (D)	19.32
2	*SETX*	c.4660T>G	p.Cys1554Gly	rs112089123	0.00584	0.0037	0.01 (D)	0.077 (B)	25.3
1	*SETX*	c.4517T>C	p.Met1506Thr	rs199974622	0.00000	0.00000	0 (D)	0.998 (D)	25.8
1	*SETX*	c.4256C>T	p.Ser1419Phe	-	-	-	0.01 (D)	0.379 (B)	-
1	*SETX*	c.4096T>C	p.Ser1366Pro	rs140147684	0.00024	0.0004	0.23 (T)	0.396 (B)	6.175
1	*SETX*	c.3809C>T	p.Pro1270Leu	rs144334281	0.00125	0.0019	0.04 (D)	0.235 (B)	23.8
1	*SETX*	c.3663G>C	p.Lys1221Asn	rs12344006	0.0042	0.0002	0.02 (D)	0.564)	21.9
1	*SETX*	c.3353C>T	p.Thr1118Ile	rs745933371	0.00000	0.00000	0.09 (T)	0.188 (B)	14.85

2	*SETX*	c.3229G>A	p.Asp1077Asn	rs145097270	0.00110	0.0012	0.06 (T)	0.17 (B)	22.6
3	*SETX*	c.3072_3074dupTGA	p.Asp1024dup	rs572772837	0.00000	0.00000	-	-	-
1	*SETX*	c.3016G>A	p.Gly1006Arg	rs141266068	0.00012	0.0001	0.4 (T)	0.003 (B)	7.307
1	*SETX*	c.2750T>C	p.Met917Thr	rs376022544	0.00011	0.0001	0.21 (T)	0.009 (B)	7.408
1	*SETX*	c.2536A>G	p.Ser846Gly	rs774820992	0.00001	0.00000	0.2 (T)	0.001 (B)	0.010
1	*SETX*	c.2416G>A	p.Asp806Asn	rs778406422	0.00002	0.00000	0.96 (T)	0 (B)	0.001
1	*SETX*	c.1468G>A	p.Val490Ile	rs763545230	0.00002	0.00000	0 (D)	0.997 (D)	23.3
1	*SETX*	c.1184C>T	p.Ser395Leu	-	-	-	0 (D)	0.999 (D)	-
2	*SETX*	c.472T>G	p.Leu158Val	rs145438764	0.00371	0.0049	0.01 (D)	0.998 (D)	23.9
1	*SETX*	c.177+3A>G	-	-	-	-	-	-	-
1	*SOD1*	c.461A>G	p.Gln154Arg	-	-	-	0.01 (D)	0.076 (B)	-
1	*SPG11*	c.7132T>C	p.Phe2378Leu	rs150571352	0.00017	0.0002	0 (D)	0.682 (P)	27.6
4	*SPG11*	c.7069C>T	p.Leu2357Phe	rs139334167	0.0008	0.0010	0.01 (D)	0.934 (D)	24.4
1	*SPG11*	c.7039T>G	p.Trp2347Gly	-	-	-	0 (D)	0.803 (P)	-
1	*SPG11*	c.6950G>A	p.Gly2317Asp	rs79186522	0.00006	0.0001	0.26 (T)	0.982 (D)	16.17
1	*SPG11*	c.6754+5G>A	-	-	-	-	-	-	-
1	*SPG11*	c.6685C>T	p.Arg2229Trp	rs751245734	0.00002	0.0000	0 (D)	0.942 (D)	28.6
1	*SPG11*	c.6419dupA	p.Ala2141GlyfsTer8	-	-	-	-	-	-
1	*SPG11*	c.6319G>A	p.Val2107Ile	rs115970214	0.00252	0.0001	0.04 (D)	0.899 (P)	22.6
1	*SPG11*	c.6100C>T	p.Arg2034Ter	rs118203963	0.00000	0.00000	-	-	41
1	*SPG11*	c.5497T>G	p.Ser1833Ala	rs766375553	0.00000	0.00000	0.1 (T)	0.817 (P)	21.7
1	*SPG11*	c.5189T>G	p.Phe1730Cys	rs755382678	0.00001	0.00000	0 (D)	0.938 (D)	27.9
1	*SPG11*	c.4923G>C	p.Lys1641Asn	rs150218102	0.00122	0.00000	0 (D)	0.978 (D)	23.3
1	*SPG11*	c.4687A>G	p.Arg1563Gly	rs75430389	0.00168	0.00000	0 (D)	0.002 (B)	24.8
1	*SPG11*	c.4365G>C	p.Trp1455Cys	rs138103656	0.00007	0.0001	0.41 (T)	0.99 (D)	19.71
1	*SPG11*	c.3930_3932delAAC	p.Thr1312del	rs759504944	0.00027	0.00000	-	-	-
1	*SPG11*	c.3551G>T	p.Ser1184Ile	rs766403944	0.00003	0.00000	0.01 (D)	0.883 (P)	25.1
1	*SPG11*	c.3019G>A	p.Val1007Ile	-	-	-	0.1 (T)	0.621 (P)	-

1	*SPG11*	c.2656T>C	p.Tyr886His	rs139687202	0.00140	0.0003	0.61 (T)	0 (B)	16.99
1	*SPG11*	c.2063T>C	p.Phe688Ser	-	-	-	0.35 (T)	0.001 (B)	-
1	*SPG11*	c.2012A>G	p.His671Arg	rs189306815	0.00007	0.0001	0.35 (T)	0.01 (B)	9.326
1	*SPG11*	c.1891+1G>T	-	rs532072204	-	-	-	-	27.7
1	*SPG11*	c.1735+3_1735+6delAAGT	-	rs312262734	-	-	-	-	-
1	*SPG11*	c.1457-4A>G	-	rs773844127	0.00010	0.0001	-	-	1.161
1	*SPG11*	c.1404G>C	p.Lys468Asn	-	-	-	0.59 (T)	0.733 (P)	-
2	*SPG11*	c.808G>A	p.Val270Ile	rs80338868	0.00609	0.0075	0.13 (T)	0.916 (D)	15.96
1	*SPG11*	c.501C>G	p.Phe167Leu	rs771462279	0.00002	0.00000	1 (T)	0.002 (B)	18.91
1	*SPG11*	c.422T>A	p.Leu141His	-	-	-	0.02 (D)	0.993 (D)	-
1	*SPG11*	c.368G>C	p.Gly123Ala	-	-	-	0.07 (T)	0.992 (D)	-
2	*SPG7*	c.184-5_184-4delTT	-	rs5818722	0.510	0.477	-	-	-
1	*SPG7*	c.908G>A	p.Gly303Glu	-	-	-	0.01 (D)	0.926 (D)	-
1	*SPG7*	c.1199G>A	p.Arg400Gln	rs541757224	0.00006	0.0001	0.09 (T)	0.977 (D)	27.4
1	*SPG7*	c.1369C>T	p.Arg457Ter	rs138671904	0.00002	0.00000	-	-	40
1	*SPG7*	c.1457G>A	p.Arg486Gln §	rs111475461	0.00452	0.0067	0.16 (T)	0.022 (B)	22.8
1	*SPG7*	c.1529C>T	p.Ala510Val §	rs61755320	0.00252	0.0037	0 (D)	0.987 (D)	25.4
1	*SPG7*	c.1611C>G	p.His537Gln	rs139952725	0.00010	0.0001	0.6 (T)	0.016 (B)	14.27
1	*SPG7*	c.2191G>A	p.Ala731Thr	rs747521455	0.00003	0.00000	0.23 (T)	0.05 (B)	24.1
1	*SPG7*	c.2299C>G	p.Gln767Glu	rs199519341	0.00001	0.00000	1 (T)	0 (B)	16.81
1	*SQSTM1*	c.88G>A	p.Glu30Lys	rs764111892	0.00000	0.00000	0.03 (D)	0.387 (B)	20.4
1	*SQSTM1*	c.98C>T	p.Ala33Val	rs200396166	0.0012	0.0018	1 (T)	0.007 (B)	18.56
1	*SQSTM1*	c.206-4C>T	-	rs370778198	0.00002	0.00000	-	-	9.929
1	*SQSTM1*	c.457G>A	p.Val153Ile	rs145056421	0.00022	0.0003	0.01 (D)	0.017 (B)	12.14
1	*SQSTM1*	c.620G>A	p.Ser207Asn	-	-	-	0.41 (T)	0.016 (B)	-
1	*SQSTM1*	c.712A>G	p.Lys238Glu	rs11548633	0.00242	0.0025	0.02 (D)	0.589 (P)	22.8
1	*SQSTM1*	c.728G>A	p.Ser243Asn	-	-	-	0.07 (T)	0.096 (B)	-
1	*SQSTM1*	c.824G>A	p.Ser275Asn	rs201923000	0.00017	0.0003	0.27 (T)	0.439 (B)	9.228

1	*SQSTM1*	c.891_905dupTGGGAATGT TGAGGG	p.Gly298_Gly302dup	rs754741710	0.00003	0.00000	-	-	-
2	*SQSTM1*	c.1005T>A	p.Asp335Glu	-	-	-	0.06 (T)	0.38 (B)	-
1	*SQSTM1*	c.1034A>T	p.Glu345Val	-	-	-	0 (D)	0.971 (D)	-
1	*SQSTM1*	c.1142C>T	p.Ala381Val	rs772122047	0.00005	0.0001	0.08 (T)	0.196 (B)	22.1
1	*SQSTM1*	c.1277C>T	p.Ala426Val	rs201239306	0.00021	0.0002	0 (D)	0.936 (D)	23.9

a Nr Subjects, number of controls carrying the variant. b dbSNP150. c Global MAF, global allele counts were calculated from all subjects in the ExAc database. d population MAF, population allele count refers to European Ancestry subject from ExAc database. §: Variant found both in ALS patients and controls. Key: MAF = minor allele frequency; SIFT: T = tolerated, D = deleterious; Polyphen: B = benign, P= possibly damaging, D = damaging; CADD scores: scaled CADD scores (Phred like) for scoring deleteriousness.

Table S4. Variants identified in this study and presence in controls.

Gene Name	cDNA Change	Protein Change	dbSNP ID[a]	ACMG	Global MAF[b]	Population MAF[c]	SIFT score	Polyphen score	Mutation Taster pred	CADD	Nr. [d] Patients	Nr. [e] Controls
**Variants found only in ALS patients**												
ALS2	c.1115C>G	p.Pro372Arg	rs190369242	3	0.0013	0.0022	0.64 (T)	0.919 (D)	0.683 (D)	19.65	1	0
BSCL2	c.844G>A	p.Ala282Thr(218)*	rs190842600	3	0.00022	0.00000	0.08 (T)	1 (D)	0.999 (D)	25.9	1	0
BSCL2	c.1033C>T	p.Arg345Trp(281)*	rs767820877	3	0.00000	0.00000	0.02(D)	0.987 (D)	0.999 (D)	21.4	1	0
BSCL2	c.689G>A	p.Ser230Asn (166)*	rs778378228	4	0.00001	0.00000	0.11 (T)	0.820 (P)	0.824 (N)	23.4	1	0
BSCL2	c.785C>T	p.Ala262Val(198)*	rs140896339	3	0.00028	0.00000	0.50 (T)	0.085(B)	0.999 (N)	18.85	1	0
BSCL2	c.1057T>A	p.Ser353Thr(189)*	rs769769807	3	0.00001	0.00000	0.17 (T)	0.006 (B)	0.999 (N)	6.03	1	0
BSCL2	c.1282C>T	p.Pro428Ser(364)*	rs369732238	3	0.00006	0.0001	0.1 (T)	0.573 (P)	0.905 (N)	18.59	1	0
BSCL2	c.1300T>C	p.Ser434Pro(370)*	rs199584887	3	0.00006	0.00000	0.44 (T)	0.001 (B)	0.999 (N)	1.58	1	0
DCTN1	c.1486G>C	p.Val496Leu	rs773897036	4	0.00002	0.00000	0.06 (T)	0.984 (D)	0.999 (D)	23.5	1	0
DYNC1H1	c.265G>A	p.Gly89Ser	rs749973847	3	0.00002	0.00000	0.74 (T)	0.019 (B)	0.999 (D)	22.8	1	0
DYNC1H1	c.3748G>A	p.Val1250Met	rs369914512	3	0.00006	0.0001	0.07 (T)	0.978 (D)	0.999 (D)	29.2	1	0
DYNC1H1	c.12213C>T[#]	p.Ile4071Ile	rs746950373	3	0.00002	0.000000	0.48 (T)	-	1 (D)	12.5	1	0
DYNC1H1	c.4183A>C	p.Lys1395Gln	-	4	-	-	0.12 (T)	1.000 (D)	0.999 (D)	34	1	0
DYNC1H1	c.5303G>T	p.Ser1768Ile	-	3	-	-	0.1 (T)	0.028 (B)	0.999 (N)	15.71	1	0
FIG4	c.1030C>A	p.Pro344Thr	-	4	-	-	0.37 (T)	0.875 (P)	0.999 (D)	25.4	1	0
FUS	c.1168+7A>G[#]	-	-	3	-	-	-	-	-	-	1	0
HSPB1	c.403T>G	p.Ser135Ala	rs766728475	4	0.00002	0.00000	0.07 (T)	0.623 (P)	0.999 (D)	25.4	1	0
HSPB3	c.347G>C	p.Arg116Pro	rs150931007	4	0.00011	0.0001	0 (D)	1 (D)	0.999 (D)	29.7	1	0
HSPB3	c.199G>A	p.Gly67Ser	rs35258119	3	0.00727	0.00000	0.2 (T)	0.036 (B)	0.999 (N)	17.28	1	0
HSPB3	c.346C>T	p.Arg116X	rs757339596	4	0.00003	0.00000	-	-	0.999 (D)	11.58	1	0
MFN2	c.1574A>G	p.Asn525Ser	rs145654854	3	0.00017	0.00021	1 (T)	0 (B)	0.753 (N)	19.62	1	0
OPTN	c.941A>T	p.Gln314Leu	rs142812715	3	0.00017	0.0003	0.01 (D)	0.999 (D)	0.993 (D)	27.7	1	0

PLEKHG5	c.3049G>A	p.Gly1017Arg	rs755699992	3	0.00000	0.00000	0.56 (T)	0.047 (B)	0.999 (N)	0.16	1	0
SETX	c.654G>C	p.Lys218Asn	rs117861188	3	0.00033	0.0006	0.0 (D)	0.961 (D)	0.900 (D)	23.8	1	0
SETX	c.59G>A	p.Arg20His	rs79740039	3	0.00683	0.01051	0.25 (T)	0.001 (B)	0.999 (N)	3.43	3	0
SETX	c.4612C>T	p.Arg1538Trp	rs147018359	3	0.00000	0.00000	0.21 (T)	0 (B)	0.999 (N)	17.31	2	0
SETX	c.3182C>T	p.Pro1061Leu	rs12352982	3	0.01604	0.00000	1 (T)	0 (B)	0.999 (N)	0.9	1	0
SETX	c.7435A>G	p.Ile2479Val	rs536912256	3	0.00006	0.00000	0.43 (T)	0.001 (B)	0.999 (N)	0.76	1	0
SETX	c.5852A>T	p.His1951Leu	-	4	-	-	0.13 (T)	0.961 (D)	0.974 (D)	25.8	1	0
SOD1	c.203T>C	p.Leu68Pro	CM110553	5	0.00000	0.00000	0.21 (T)	0.001 (B)	0.99 (N)	6.15	1	0
SOD1	c.217G>A	p.Gly73Ser	rs121912455	5	0.00000	0.00000	0.0 (D)	0.970 (D)	0.999 (D)	29.3	1	0
SPG11	c.1348A>G	p.Ile450Val	rs3759873	1	0.01672	0.0045	0.78 (T)	0 (B)	0.994 (D)	6.31	1	0
SPG11	c.3037A>G	p.Lys1013Glu	rs111347025	2	0.00861	0.01448	0.73 (T)	0.002 (B)	0.963 (D)	22.7	2	0
SPG11	c.6224A>G	p.Asn2075Ser	rs140824939	3	0.0031	0.00489	0.49 (T)	0 (B)	0.999 (N)	0.28	1	0
SPG11	c.5986_5987insT	p.Cys1996Leufs*4	rs312262775	5	0.00002	0.00000	-	-	-	-	1	0
SPG11	c.1675T>A	p.Ser559Thr	rs773680273	3	0.00001	0.00000	0.2 (T)	0.990 (D)	0.591 (D)	20.8	1	0
SPG11	c.2764G>A	p.Val922Ile	rs139399250	3	0.00006	0.0001	0.43 (T)	0.002(B)	0.999 (N)	2.25	1	0
SPG11	c.6201A>T[#]	p.Gly2067Gly	rs764991726	3	0.00001	0.00000	1 (T)	-	0.987 (D)	7.48	1	0
SPG11	c.4826delT	p.Met1609Serfs*31	-	4	-	-	-	-	1 (D)	8.99	1	0
SQSTM1	c.352C>T	p.Pro118Ser	rs200152247	3	0.00006	0.0001	0.54 (T)	0.009 (B)	0.999 (D)	20.9	1	0
SQSTM1	c.802C>G	p.Leu268Val	rs753685955	3	0.00001	0.00000	1 (T)	0.004 (B)	0.824 (N)	17.73	1	0
TBK1	c.1190T>C	p.Ile397Thr	rs755069538	5	0.0001	0.0003	0.31 (T)	0.039 (B)	0.999 (D)	22.8	1	0
**Variants found in ALS patients and controls (frequency from 0.15% to 1%)**												
ALS2	c.4119A>G	p.Ile1373Met	rs61757691	3	0.00291	0.0049	0.05 (D)	0.439 (B)	0.999 (D)	21.9	1	1
ALS2	c.3206G>A	p.Gly1069Glu	rs200706696	3	0.00053	0.00065	0.0 (D)	1 (D)	1 (D)	27	1	1
ERBB4	c.1122T>G	p.His374Gln	rs76603692	4	0.00133	0.00187	0.35 (T)	0.189 (B)	0.996 (D)	17.24	2	5
FIG4	c.122T>C	p.Ile41Thr	rs121908287	4	0.00105	0.00167	0 (D)	1 (D)	0.999 (A)	25.8	1	2
FUS	c.681_689delCG GCGGTGG	p.Gly227_Gly229del	-	3	-	-	-	-	-	-	1	4

MFN2	c.1403G>A	p.Arg468His	rs138382758	4	0.00194	0.00281	0.07 (T)	0.787 (P)	0.999 (A)	23.3	1	3
PLEKHG5	c.319C>T	p.Arg107Cys	rs111400494	3	0.00283	0.00479	0.01(D)	0.49 (P)	0.999 (D)	24.6	1	1
SPG7	c.1457G>A	p.Arg486Gln	rs111475461	3	0.00589	0.01031	0.27 (T)	0.077 (B)	0.999 (D)	22.8	1	1
SPG7	c.1529C>T	p.Ala510Val	rs61755320	4	0.00311	0.00458	0 (D)	1 (D)	0.999 (D)	25.4	2	1
**Variants found in ALS patients and controls (frequency > 1%)**												
BSCL2	c.1280T>C	p.Leu427Pro (363)*	rs145649423	3	0.00211	0.0035	0.26 (T)	0.008 (B)	0.997 (D)	21.2	3	9
ERBB4	c.882A>G[#]	p.Pro294Pro	rs77309171	3	0.00316	0.00531	1 (T)	-	1 (D)	13.5	1	8

a dbSNP150. b Global MAF, global allele counts were calculated from all subjects in the ExAc database. c population MAF, population allele count refers to European Ancestry subject from the ExAc database. d Nr. Patients, number of ALS patients carrying the variant. e Nr. Controls, number of controls carrying the variant. Key: American College of Medical Genetics and Genomics (ACMG) Classification: 1 = benign; 2 = likely benign; 3 = uncertain significance; 4 = likely pathogenic; 5 = pathogenic; MAF = minor allele frequency; SIFT: T = tolerated, D = deleterious; Polyphen: B= benign, P= possibly damaging, D = damaging; Mutation Taster : D = disease causing, N= polymorphism, A = disease causing automatic; CADD scores: scaled CADD scores (Phred like) for scoring deleteriousness.

Table S5. Overview of variants identified in ALS patients and controls.

Gene	ALS Patients (83)		Non-neurological Controls (332)		Chi-Square
	Total Variants (Different Variants)[a]	% Variants/ Total Nr. Alleles[b]	Total Variants (Different Variants)[a]	% Variants/ Total Nr. Alleles[b]	*p*-value[c]
**ALS genes**					
ALS2	3 (3)	1.81	14 (14)	2.11	0.806
DCTN1	1 (1)	0.60	14 (9)	2.11	0.192
DYNC1H1	4 (4)	2.41	11 (10)	1.66	0.528
ERBB4	3 (2)	1.81	9 (5)	1.36	0.662
FIG4	2 (2)	1.20	4 (3)	0.60	0.412
FUS	2 (2)	1.20	8 (5)	1.20	0.997
OPTN	1 (1)	0.60	5 (5)	0.75	0.837
PLEKHG5	2 (2)	1.20	40 (23)	6.02	0.0130
SETX	9 (6)	5.42	30 (21)	4.52	0.622
SOD1	2 (2)	1.20	1 (1)	0.15	0.043
SPG7	3 (2)	1.81	10 (9)	1.51	0.779
SPG11	8 (7)	4.82	32 (28)	4.82	1
SQSTM1	2 (2)	1.20	14 (13)	2.11	0.449
TBK1	1 (1)	0.60	0 (0)	0	-
**Genes associated with other MNDs /HMN/dSMA/CMT2**					
BSCL2	10 (8)	6.02	14 (7)	2.11	0.007
HSPB1	1 (1)	0.60	5 (3)	0.75	0.778
HSPB3	3 (3)	1.81	1 (1)	0.15	0.006
MFN2	2 (2)	1.20	11 (9)	1.66	0.675

a. Number of ALS Patients/Controls carrying a variant (Number of different variants found). b. All variants found were heterozygous. Percentages have been made on 166 alleles (ALS patients) and 664 alleles (controls). c. Chi-square statistic: The result is considered significant with p value < 0.003 (p value < 0.05 adjusted with Bonferroni correction).

Table S6. Cox proportional hazards regression multivariate analysis on survival and time to King stage 4 (MAF < 0.001)

	Survival		Time to King stage 4	
**Factor**	**HR (95% CI)**	***p* Value**	**HR (95% CI)**	***p* Value**
Rare variants (n)		<0.001		0.001
0	1		1	
1	2.15 (1.04–4.44)	0.038	0.937 (0.45–1.92)	0.94
2	6.18 (2.60–14.65)	<0.001	4.76 (2.06–11.03)	<0.001
Site of symptoms onset				
Spinal	1	0.029	ns	ns.
Bulbar	2.32 (1.09–4.93)			
ALSFRS-R decline (points/month)				
≤ 0.60	1	<0.004	1	0.045
> 0.60	4.02 (1.55–10.40)		2.69 (1.02–7.06)	
Diagnostic delay (months)				
≤ 10	ns	ns	ns	ns
> 10				
Dementia				
No	ns	ns	ns	ns
Yes				
Age at the onset (years)				
22–59	ns	ns	1	0.003
>60			2.86 (1.44–5.70)	

Variables included in the model: age at onset (22–59, >60); Presence of dementia (yes, no); Site of symptoms onset (bulbar; spinal); ALSFRS-R decline (≤0.60, >0.60 points/months); Diagnostic delay (≤10 months; >10months); MAF (0, 1, 2 gene variants). ALSFRS-R= ALS Functional Rating Scale revised; MAF = minor allele frequency; HR = hazard ratio; CI = confidence interval; ns = not significant

Table S7. Genes present in the panel

Gene	Gene name	Chromosome	Accession Number (NM)	Accession Number (NP)	Coding exons	Protein (aa)	OMIM number	Phenotype OMIM number	Disease	Inheritance
ALS genes										
ALS2	Alsin Rho guanine nucleotide exchange factor	2q33.1	NM_020919	NP_065970	33	1,657	606352	205100; 606353; 607225	ALS2, Primary lateral sclerosis, juvenile; Spastic paralysis, infantile onset ascending	AR; AR; AR
ANG	Angiogenin	14q11.2	NM_001145	NP_001136	1	147	105850	611895	ALS9	AD
DCTN1	Dynactin subunit1	2p13.1	NM_004082	NP_004073	32	127	601143	607641; 168605; 105400	Neuropathy, distal hereditary motor, type VIIB; Perry syndrome; Amyotrophic lateral sclerosis, susceptibility	AD; AD; AD/AR
DYNC1H1	Dynein cytoplasmic 1 heavy chain 1	14q32.31	NM_001376	NP_001367	78	4,646	600112	614228; 614563; 158600	Charcot-Marie-Tooth disease, axonal, type 20; Mental retardation, autosomal dominant 13; Spinal muscular atrophy, lower extremity-predominant 1, AD	AD; AD; AD
ERBB4	Erb-b2 receptor tyrosine kinase 4	2q34	NM_005235	NP_005226	28	1,308	600543	615515	ALS19	AD
FIG4	FIG4 phosphoinositide 5-phosphatase	6q21	NM_014845	NP_001138600	23	907	609390	612691; 612577; 611228; 216340	Polymicrogyria, bilateral temporooccipital; ALS11; CMT4J; Yunis-Varon syndrome	AR; AD; AR; AR
FUS	FUS RNA binding protein	16p11.2	NM_004960	NP_004951	15	526	137070	608030; 614782	ALS6, with or without frontotemporal dementia; Tremor, hereditary essential, 4	AD/AR; AD
GARS	Glycyl-tRNA synthetase	7p14.3	NM_002047	NP_002038	17	739	600287	601472; 600794	CMT2D; Neuropathy, distal hereditary motor, type VA	AD; AD
OPTN	Optineurin	10p13	NM_001008211	NP_001008212	13	577	602432	613435; 137760; 606657	ALS12; Glaucoma 1, open angle, E; Glaucoma, normal tension, susceptibility	AD/AR, AD; AD/AR
PFN1	Profilin 1	17p13.2	NM_005022	NP_005013	3	140	176610	614808	ALS18	AD
PLEKHG5	Pleckstrin homology and RhoGEF domain containing G5	1p36.31	NM_198681	NP_941374	22	1,083	611101	615376; 611067	Charcot-Marie-Tooth disease, recessive intermediate C; Spinal muscular atrophy, distal, autosomal recessive, 4	AR; AR; AR

SETX	Senataxin	9q34.13	NM_015046	NP_055861	24	2,677	608465	602433; 606002	ALS4, Spinocerebellar ataxia, autosomal recessive 1	AD; AR
SOD1	Superoxide dismutase 1	21q22.11	NM_000454	NP_000445	5	154	147450	105400	ALS1	AD; AR
SPAST	Spastin	2p22.3	NM_014946	NP_055761	17	6,161	604277	182601	SPG4	AD
SQSTM1	Sequestosome 1	5q35.3	NM_003900	NP_003891	8	440	601530	616437; 617158; 617145; 167250	FTD and/orALS3; Myopathy, distal, with rimmed vacuoles; Neurodegeneration with ataxia, dystonia, and gaze palsy, childhood-onset; Paget disease of bone 3	AD; AD; AR; AD
SPG7	Paraplegin matrix AAA peptidase subunit	16q24.3	NM_003119	NP_003110	17	795	602783	607259	SPG7	AD; AR
SPG11	Spatacsin vesicle trafficking associated	15q21.1	NM_025137	NP_079413	40	2,443	610844	602099; 616668; 604360	ALS5, CMT2X, HSPG11	AR
TARDBP	TAR DNA binding protein	1p36.22	NM_007375	NP_031401	5	414	605078	612069; 612069	ALS10, with or without FTD; Frontotemporal lobar degeneration, TARDBP-related	AD; AD
TBK1	TANK-binding Kinase 1	12q14.2	NM_013254.3	NP_037386.1	21	729	604834	616439; 617900	FTD, ALS4, Encephalopathy, acute, infection-induced(herpes-specific), susceptibility to 8	AD; AD
UBQLN2	Ubiquilin 2	Xp11.21	NM_013444	NP_038472	1	624	300264	300857	ALS15, with or without FTD	XLD
VAPB	VAMP associated protein B and C	20q13.32	NM_004738	NP_004729	6	243	605704	608627; 182980	ALS8; Spinal muscular atrophy, late-onset, Finkel type	AD; AD
VCP	Valosin containing protein	9p13.3	NM_007126	NP_009057	17	8,069	601023	613954; 616687; 167320	ALS14, with or without FTD; CMT2Y; Inclusion body myopathy with early-onset Paget disease and frontotemporal dementia 1	AD; AD; AD
**Genes associated with other MNDs /HMN/dSMA/CMT2**										
BSCL2	BSCL2, seipin lipid droplet biogenesis associated	11q12.3	NM_001122955	NP_001116427	11	462	606158	600794; 270685; 615924; 269700	Neuropathy: distal hereditary motor, type VA; Silver spastic paraplegia syndrome; Encephalopathy, progressive, with or without lipodystrophy; Lipodystrophy, congenital generalized, type 2	AD; AD; AR; AR
HSPB1	Heat shock protein family B (small) member 1	7q11.23	NM_001540	NP_001531	3	205	602195	606595; 608634	CMT2F; Neuropathy, distal hereditary motor, type IIB	AD; AD

HSPB3	Heat shock protein family B (small) member 3	5q11.2	NM_006308	NP_006299	1	150	604624	613376	Neuronopathy, distal hereditary motor, type IIC	AD
HSPB8	Heat shock protein family B (small) member 8	12q24.23	NM_014365	NP_055180	3	196	608014	608673; 158590	CMT2L; Neuropathy, distal hereditary motor, type IIA	AD; AD
MFN2	Mitofusin 2	1p36.22	NM_014874	NP_055689	17	757	608507	609260; 617087; 601152	CMT2A; CMT2B; Hereditary motor and sensory neuropathy VIA	AD; AR; AD
TRPV4	Transient receptor potential cation channel subfamily V member 4	12q24.11	NM_001177431	NP_001170902	15	871	605427	617383; 113500; 606835; 606071; 156530; 168400; 181405; 184095; 600175; 184252; 613508	Avascular necrosis of femoral head, primary, 2; Brachyolmia type 3; Digital arthropathy-brachydactyly, familial; Hereditary motor and sensory neuropathy, type Iic; Metatropic dysplasia; Parastremmatic dwarfism; Scapuloperoneal spinal muscular atrophy; SED, Maroteaux type; Spinal muscular atrophy, distal, congenital nonprogressive; Spondylometaphyseal dysplasia, Kozlowski type	AD; AD; AD; AD; AD; AD; AD; AD; AD; AD

Key: ALS = amyotrophic lateral sclerosis; MND = motor neuron disease; FTD= frontotemporal dementia; OMIM = Online Mendelian Inheritance in Man; AD = autosomal dominant; AR = autosomal recessive; XLD = X-linked dominant; CMT = Charcot-Marie-Tooth; SPG = spastic paraplegia.

Table S8. Criteria for evaluation and filtering of the identified variants in this study

**Filtering strategy**
1. Variants in coding regions, including flanking intronic regions (10 flanking bases), 5′ and 3′ UTR regions
2. Coverage depth >30x
3. Qscore ≥ 30
4. Visual examination of the genetic data by IGV
5. MAF < 0.01 in variant databases: dbSNP150,ExAC
6. Exclusion of synonymous variants exception for those located in or near splice site
7. Sanger confirmation

Key: IGV= Integrative Genome Viewer; ExAC=Exome Aggregation Consortium Sequencing Project

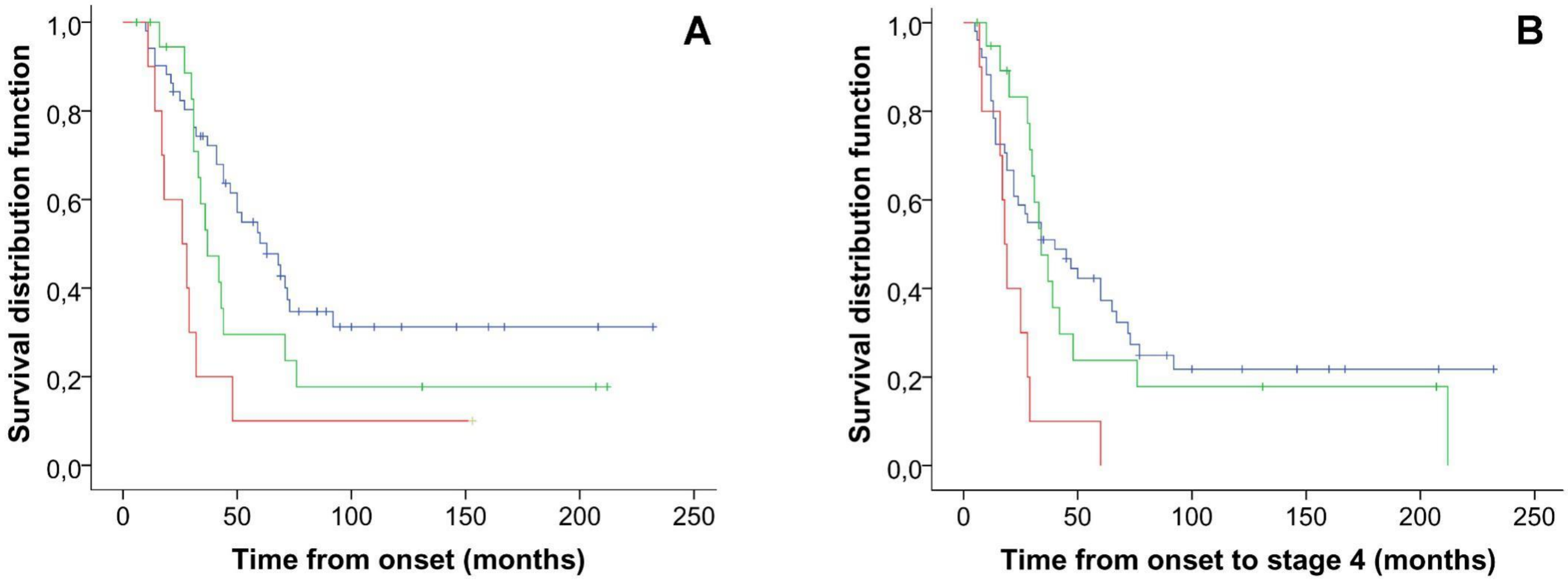

Figure S1: Kaplan-Meier univariate analysis for Survival (**A**) and time to King's stage 4 (**B**) (MAF< 0.001). Blue line: patients harboring no variants; green line: one rare variant; red line: two or more rare variants.

These results of Kaplan-Meier analysis were confirmed at MAF < 0.001 in the European population of the ExAC database. Kaplan-Meier analysis showed that higher variant burden is associated with reduced survival in our cohort of ALS patients, log-rank (Mantel-Cox) $\chi2$ = 10.61, p = 0.005 (Figure S1). ALS patients harboring two or more rare variants had a significantly shorter median survival (26.00 months, 95% CI 10.5–41.5 months) compared with ALS patients carrying one rare variant (37.00 months, 95% CI 26.3–47.7) and with ALS patients harboring no rare variants (63.00 months, 95% CI 44.0 - 82.0 months). We also confirmed that higher rare variant burden is associated with reduced time to reach King stage 4, log-rank (Mantel-Cox) $\chi2$=8.89, p=0.01 (Figure 2B) compared with ALS patients harboring one or no rare variants. ALS patients harboring two or more rare variants have a significantly shorter median time to King stage 4 (18.00 months, 95% CI 14.9 - 21.1 months) compared with ALS patients carrying one rare variant (34.00 months, 95% CI 26.0–42.0) and with ALS patients harboring no rare variants (40.00 months, 95% CI 17.7–62.3 months).
